# Mechanisms underlying the loss of migratory behaviour in a long‐lived bird

**DOI:** 10.1111/1365-2656.70035

**Published:** 2025-04-02

**Authors:** Pedro Andrade, Aldina M. A. Franco, Marta Acácio, Sandra Afonso, Cristiana I. Marques, Francisco Moreira, Miguel Carneiro, Inês Catry

**Affiliations:** ^1^ CIBIO, Centro de Investigação em Biodiversidade e Recursos Genéticos, InBIO Laboratório Associado Campus de Vairão, Universidade do Porto Vairão Portugal; ^2^ BIOPOLIS Program in Genomics, Biodiversity and Land Planning, CIBIO Vairão Portugal; ^3^ School of Environmental Sciences University of East Anglia Norwich Norfolk UK; ^4^ School of Zoology Faculty of Life Sciences, Tel Aviv University Tel Aviv Israel; ^5^ Departamento de Biologia Faculdade de Ciências, Universidade do Porto Porto Portugal; ^6^ CIBIO, Centro de Investigação Em Biodiversidade e Recursos Genéticos, InBIO Laboratório Associado, Instituto Superior de Agronomia Universidade de Lisboa Lisbon Portugal; ^7^ Centre for Ecology, Evolution and Environmental Changes (CE3C) & CHANGE ‐ Global Change and Sustainability Institute Faculdade de Ciências da Universidade de Lisboa Lisbon Portugal

**Keywords:** adaptation, bird migration, developmental plasticity, generational shifts, GPS‐tracking, ontogeny, phenotypic flexibility, white stork

## Abstract

1. Human‐induced environmental changes are changing the migration patterns of birds worldwide. Species are adjusting migration timing, shortening and diversifying migratory routes or even transitioning towards residency. While the ultimate causes driving changes in migratory patterns are well established, the underlying mechanisms by which migratory species adapt to environmental change remain unclear.
2. Here, we studied the mechanisms driving the recent and rapid loss of migratory behaviour in Iberian white storks *Ciconia ciconia*, a long‐lived and previously fully migratory species through the African–Eurasian flyway. We combined 25 years of census data, GPS‐tracking data from 213 individuals (80 adults and 133 first‐year juveniles) tracked up to 7 years and whole‐genome sequencing to disentangle whether within‐ (phenotypic flexibility) or between‐ (developmental plasticity or microevolution, through selection) individual shifts in migratory behaviour over time explain the observed population‐level changes towards residency.
3. Between 1995 and 2020, the proportion of individuals no longer migrating and remaining in Southern Europe year‐round increased dramatically, from 18% to 68–83%. We demonstrate that this behavioural shift is likely explained by developmental plasticity. Within first‐year birds, 98% crossed the Strait of Gibraltar towards their African wintering grounds, in Morocco or Sub‐Saharan Africa. However, the majority shifted towards a non‐migratory strategy as they aged—the proportion of migrants decreased to 67% and 33%, in their second and third year of life, respectively. Supporting these findings, only 19% of GPS‐tracked adults migrated. We did not find evidence of phenotypic flexibility, as adults were highly consistent in migratory behaviour over multiple years (only 3 individuals changed strategy between years, out of 113 yearly transitions), nor of selection acting on genetic variation, since genomes of adult migrants and residents are essentially undifferentiated and we did not find evidence of selective sweeps in resident birds.
4. Our results suggest that through developmental plasticity, traits that are plastic during specific windows of development become fixed during adulthood. Thus, inter‐generational shifts in the frequency of migratory and non‐migratory young individuals could drive population changes in migratory behaviour. This can provide a mechanism for long‐lived migratory birds to respond to rapid human‐driven environmental changes.

## Graphical abstract

White stork populations in Iberia are undergoing a profound change in their migratory patterns, becoming mostly resident. By combining census data, GPS‐tracking and genomic analyses, Andrade et al. link this behavioural change to inter‐generational shifts in the frequency of migratory and non‐migratory young individuals (likely associated with developmental plasticity).

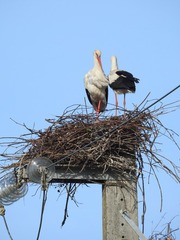

## 1 INTRODUCTION

The migratory behaviour of many bird species is shifting in response to human‐induced environmental change. This includes changes in migratory phenology, in the limits of breeding and wintering ranges, the shortening of migration distances or even the disruption of migration (Able & Belthoff, 1998; Berthold et al., 1992; Curley et al., 2020; Horton et al., 2020; Rushing et al., 2020; Visser et al., 2009). While the ultimate causes driving observed changes in migratory patterns are well established (e.g. human‐driven climate change), the mechanisms by which migratory species adapt to environmental change remain largely unknown (Åkesson & Helm, 2020; Charmantier & Gienapp, 2014). At the population level, changes in avian migratory behaviour can occur through three distinct but not mutually exclusive processes: (i) phenotypic flexibility, induced by environmental conditions, which can be reversible and reflects within‐individual changes in the expression of a phenotype (Charmantier et al., 2008); (ii) evolution, through longer term, inter‐generational shifts in the frequency of alleles controlling migratory behaviour (Berthold et al., 1992); and (iii) developmental plasticity, an irreversible phenotypic change induced by environmental, physiological or social conditions juveniles experience during ontogeny (Piersma & Drent, 2003), leads to population‐level changes driven by inter‐generational shifts, as young individuals, with the new phenotype, replace older ones in the population over generations.

Cross‐breeding experiments in passerines, conducted in captivity, indicate substantial heritability of migratory traits (Pulido et al., 2001; Pulido & Berthold, 2010). Heritable differences in migratory strategies have been tentatively linked to either large genomic regions of high differentiation, likely harbouring chromosomal inversions containing dozens to hundreds of genes (Delmore et al., 2016; Lundberg et al., 2017; Sanchez‐Donoso et al., 2022), or to selection operating on genes that are potentially linked to spatial behaviour and learning (Delmore et al., 2020; Gu et al., 2021; Toews et al., 2019). Although these examples help to illustrate how genetic processes may lead to changes in migratory behaviour through genetics, plasticity could provide a faster mechanism for rapid acclimation to environmental change (Åkesson & Helm, 2020; Both & Visser, 2001; Charmantier et al., 2008; Charmantier & Gienapp, 2014; Dias et al., 2011; Horton et al., 2023; Teitelbaum et al., 2016; Teplitsky et al., 2008), which is particularly relevant for long‐lived species since important environmental changes can occur within the lifetime of individuals.

Attempts to disentangle the relative roles of different modes of plasticity in the evolution of bird migration are hampered by the difficulty of collecting long‐term population‐wide information, as well as repeated individual data through periods of shifts in migratory behaviour at the population level (Conklin et al., 2021; Fraser et al., 2019; Gill et al., 2019). Recently, developments in tracking technology warranted to bridge this knowledge gap are becoming more widely available (Åkesson & Helm, 2020). However, recent research using repeat‐tracking data does not unambiguously support a single mechanism. Some studies have shown that phenotypic flexibility can largely account for population‐level advances in migration timing of long‐distance migrants (e.g. Conklin et al., 2021). Yet, a growing number of studies document high within‐individual levels of repeatability in migratory timing, routes and wintering sites over multiple years (Carneiro et al., 2019; Pedersen et al., 2018; Stanley et al., 2012; Vardanis et al., 2011) This suggests that, for species with low individual flexibility, inter‐generational shifts through developmental plasticity could drive population‐level changes in migratory behaviour in a few generations, with young individuals being the agents of such change (Gill et al., 2019; Verhoeven et al., 2018, 2021). These studies, however, have not explicitly discounted the effects of underlying genetic variation. Long‐lived birds, in particular, have extended developmental periods in which young birds can learn from past experiences and improve their migratory performance (Campioni et al., 2020; Sergio et al., 2014). Moreover, in social species, individuals can alter their migratory behaviour based on social learning from conspecifics, also contributing to population‐level shifts in migratory behaviour (Mueller et al., 2013; Teitelbaum et al., 2016).

**FIGURE 1:**
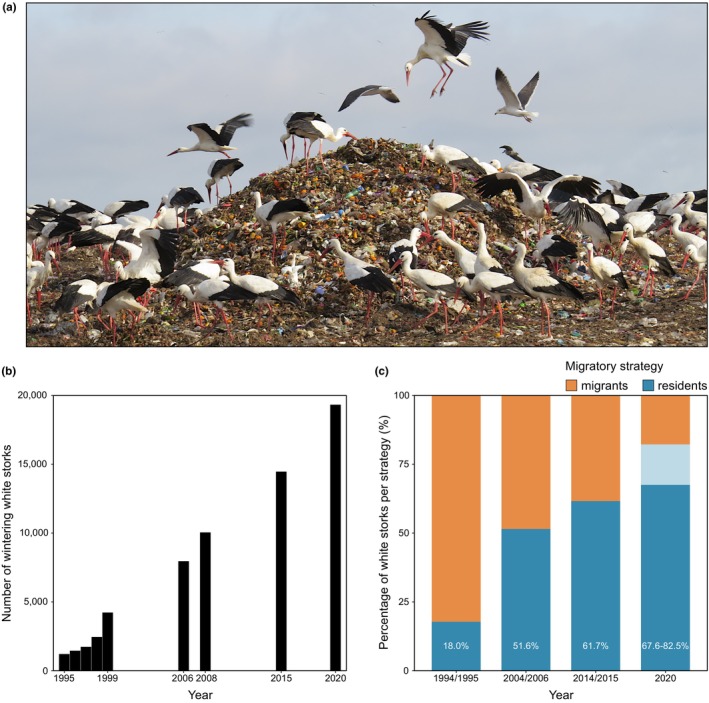
Loss of migratory behaviour in the white stork *Ciconia ciconia*. (a) Increased food availability, particularly at landfills, is one of the major factors promoting an increase in the wintering population of white storks in Europe (Catry et al., 2017; Cheng et al., 2019; Flack et al., 2016). (b) Population trends of wintering white storks from the Portuguese population between 1995 and 2020 (Catry et al., 2017, and this study). (c) Changes in the percentage of resident white storks in Portugal in the last 25 years. The proportion of residents was estimated as the ratio between the number of breeding and wintering individuals (see Section 2). To estimate the proportion of residents in 2020 (no breeding census was performed since 2014), we used the number of breeding pairs in 2014 (minimum value) and the predicted number of breeding pairs given by the linear model (*y* = 838.9*x* + 5890.1) using the data available from the previous census (maximum value). The range of this estimate is represented by a lighter shade of blue.

Many studies investigating recent changes in avian migratory patterns have focused mostly on shifts in routes or timing of migration (time and space). However, even more profound changes have been recorded, with individuals abandoning migration completely in previously partially or fully migratory populations and establishing non‐migratory populations (Berthold, 2001; Newton, 2008; Van Vliet et al., 2009), a process which in the Eurasian blackcap *Sylvia atricapilla* is likely driven by selection on genetic variation (De Zoeten & Pulido, 2020; Pulido & Berthold, 2010). While these changes can dramatically affect species distributions and ecosystems with unforeseen conservation challenges (Gill et al., 2019), the mechanisms underlying the rapid losses of migratory behaviour are still poorly understood. Here, we combine two decades of census data, movement information from 213 birds that were GPS‐tracked for up to 7 years and whole‐genome sequencing to investigate the loss of migratory behaviour in the white stork (*Ciconia ciconia*, Figure 1a). This long‐lived species is an iconic symbol for long‐distance migrations in many European countries, where juveniles and adults migrate together in mixed flocks (Rotics et al., 2016), as naïve birds require guidance from older and more experienced individuals to successfully reach their wintering grounds (Dallinga & Schoenmakers, 1987). In recent decades, however, a growing number of individuals no longer carry out their annual autumn migration from Europe to Africa, remaining in Southern Europe all year‐round (Archaux et al., 2004; Cheng et al., 2019; Flack et al., 2016; Rotics et al., 2017). Increased food availability from anthropogenic sources, including food waste at landfills (Gilbert et al., 2016; Soriano‐Redondo et al., 2021; Tortosa et al., 2002) and the invasive red crayfish *Procambarus clarkii* (Ferreira et al., 2019), together with increases in winter temperatures (Schulz & Schulz, 1999) are thought to have contributed to the suppression or shortening of migration in European white storks. Nonetheless, the mechanisms underlying this behavioural shift at the population level remain unknown.

National surveys performed during the last 25 years (Catry et al., 2017, and additional data from the present study) show that the number of Portuguese white storks remaining in the country during the winter increased 16‐fold from 1187 individuals in 1995 to 19,295 in 2020 (Figure 1b), coinciding with a northward wintering area range expansion (Figure S1). The breeding population increased 3.5 times in the same period (from 3302 in 1994 to 11,691 breeding pairs in 2014; Encarnação, 2015), indicating a steep rise in the proportion of resident (i.e. non‐migratory) individuals, from 18% to 68%–83% (Figure 1c). Here, we aim to understand the mechanisms driving the observed population‐level changes in the migratory behaviour of white storks. Specifically, we investigate whether such changes can derive from: (i) within‐individual reversible changes in the expression of the phenotype (phenotypic flexibility); (ii) between‐individual, non‐reversible changes in the expression of the phenotype, linked to developmental effects (developmental plasticity); and (iii) selection on genetic variation (microevolution).

## 2 MATERIALS AND METHODS

### 2.1 Population trends of resident white storks

White stork national censuses have been carried out in Portugal over the last 25 years to monitor the species population trends following declines that occurred earlier during the 20th century. Breeding censuses were performed in 1994, 2004 and 2014 by quantifying the number of occupied nests in spring (Encarnação, 2015). Non‐breeding censuses were conducted from mid‐September to early October from 1995 to 1999 (annually) and in 2006, 2008, 2015 (Catry et al., 2017) and 2020 (this study) and included all areas where the species is known to winter regularly, particularly areas of high food availability during winter, such as landfill sites and rice fields, where storks tend to concentrate (Catry et al., 2017). The census period was chosen because most migratory individuals cross the Strait of Gibraltar towards their African wintering grounds between July and early September (Acácio et al., 2022; Fernández‐Cruz, 2005; Soriano‐Redondo et al., 2020), and the pre‐nuptial return migration to the breeding areas only starts in November (Bécares et al., 2019, authors' tracking data). Moreover, from mid‐September to mid‐October, the number of immigrants is very small, as suggested by tracking data and ring resights of non‐Portuguese individuals (Supplementary text, Figure S2). Thus, all birds counted during the non‐breeding surveys were considered as residents (see Supplementary text for a more detailed description). For further validation, we estimated the proportion of resident birds using data from our GPS‐tracked adult population (see below) between 2016 and 2022 and compared it with the estimates from the national surveys.

### 2.2 GPS tracking of white storks

We captured 213 white storks (80 adults and 133 juveniles) in southern Portugal between 2016 and 2022 to deploy GPS‐tracking devices (Table S1). Storks were tracked for a minimum of 2 months and up to 7 years, enough to identify at least one migratory strategy per individual. Adult white storks were captured either at their nests, using remotely activated clap nets or at landfill sites using nylon leg nooses. Juveniles were taken from the nests for tag deployment and returned afterwards. All captured individuals were fitted with individually coded rings, measured and blood samples were collected and stored in ethanol. GPS/GSM loggers (‘Flyway‐50’ from Movetech Telemetry, ‘Ornitrack‐50’ from Ornitela and 50 g bird solar tags from e‐obs GmbH’) were mounted on the back of the birds as backpacks with a Teflon harness; the total weight was 50–90 g, 1.5%–3.7% of the birds' body mass. The loggers were programmed to record GPS positions every 20 min. The procedure was approved by Instituto da Conservação da Natureza e das Florestas (Portugal; permits 493/2016/CAPT, 662/2017/CAPT, 549/2018/CAPT, 248/2019/CAPT, 365/2020/CAPT, 199/2021/CAPT and 542/2022/CAPT).

Migrants were defined as birds that crossed the Strait of Gibraltar (to Morocco or Sub‐Saharan Africa), while residents remained in Iberia all year‐round (Portugal and Spain). This classification is based on the discontinuity of migration distances travelled by the two groups (Figure S3) and the assumption that individuals following either the migratory or resident strategy face challenges that are more similar within their own group than across the different strategies. The Strait of Gibraltar is a recognized bottleneck for birds migrating to and from Europe and Africa and a very demanding stage along the migratory pathway. The conditions experienced during sea crossing may have deleterious carry‐over effects, as happens with other barriers like the Sahara Desert (Mellone, 2020). Irrespective of the wintering grounds of the migrants (North or Sub‐Saharan Africa), all storks that crossed the Strait of Gibraltar did so during the peak of the migration season (August–October).

### 2.3 Ontogeny and consistency in migratory behaviour of white storks

To investigate shifts in migratory decisions of white storks with age, we fitted a binomial generalized linear mixed model (g*lmer* function in the *R‐*package *lme4*; Bates et al., 2014) with migration strategy (resident or migrant) and age (*n* = 133, 24, 12 and 83 individuals with 1, 2, 3 and ≥4 years) as the response and explanatory variables, respectively, and bird ID as a random effect. Contrary to juveniles, birds tagged as breeding adults (≥4 years old) entered the model only once. Thus, three adults that changed migratory strategy were not included in the analysis.

To assess the degree of individual consistency (or conversely, flexibility) in the choice of the wintering latitude (and thus, on migratory strategy), we used data from adults and juveniles tracked in multiple years. For each bird, we determined the wintering latitude as the minimum latitude reached in October, as by then white storks are in their main wintering grounds and do not show significant dispersive or migratory movements (Rosa et al., 1998). Among adults, 48 individuals were tracked for at least two consecutive winters; 11, 17, 15, 3, 1 and 1 storks were repeatedly tracked through two to seven winters, respectively (161 bird‐winter comparisons; Table S1). Within juveniles, we tracked 24 juveniles for at least two consecutive winters; 13, 5, 3, 1, 1, and 1 storks were repeatedly tracked for two to seven winters, respectively. However, to estimate individual repeatability among juveniles, we discarded bird‐winter after the age of first breeding (3–4 years; remaining with 59 bird‐winter comparisons, Table S1). Repeatability of wintering latitudes was analysed using the *rpt* function for Gaussian data in the *R*‐package *rptR* (Stoffel et al., 2017).

### 2.4 Reference genome assembly

We assembled the genome of the white stork using linked read technology (10X Genomics, San Francisco, USA). Snap‐frozen fresh blood from a female bird (metal ring number MR09149, CEMPA), over‐wintering within the Iberian Peninsula, was used for high‐molecular weight DNA extraction using a salt‐based protocol (Enbody et al., 2021). Prior to library preparation, DNA quantity and integrity were assessed using a NanoDrop instrument, Qubit dsDNA BR Assay Kit and Agilent Genomic DNA ScreenTape (Agilent). The average size of the extracted DNA was estimated to be above 60 kb. A chromium library was prepared by Novogene UK following the manufacturer's instructions and sequenced on an Illumina instrument using 2 × 150 bp paired‐end reads. This produced a total of 510,084,146 reads (corresponding to an effective coverage of 60.9×). Chromium sequencing data are available in the Sequence Read Archive under BioProject PRJNA713582.

To assemble the genome using linked‐read data, we used *Supernova* v2.0 (Weisenfeld et al., 2017) with default parameters. A preliminary version of the assembly subjected to an automated Contamination Screen from NCBI's genome submission portal identified a number of sequences that were either duplicated, of foreign origin or mitochondrial, and these were removed from the assembly. Adaptor sequences (NGB01088.1, NGB01096.1, NGB01047.1 and NGB01039.1) were also identified flanking gaps in 20 independent regions of the assembly, suggestive of local misassemblies. To test for this, we mapped the whole genome re‐sequencing dataset from 54 birds (see below) to the preliminary assembly and used *IGV* v2.8.2 (Thorvaldsdóttir et al., 2013) to look for pairs of reads spanning each gap. Nineteen of 20 of these regions were considered to result from correct assembly, since for each of them, we found at least 10 pairs of reads that respected the following criteria: (1) each read mapped on a different side of the gap; (2) pairs of reads had mapping quality 60; and (3) reads had no supplementary or secondary alignments. Following this inspection, adaptor sequences were hard‐masked. For the remaining region, a large gap meant it was not possible to rule out a misassembly, so we split the scaffold into two smaller ones.

To evaluate the quality of our final assembly, we calculated summary metrics with the script *assemblathon_stats.pl* (https://github.com/KorfLab/Assemblathon; Bradnam et al., 2013). We also quantified the number of highly conserved single‐copy orthologues using *BUSCO* v5 (Simão et al., 2015). For *BUSCO* analysis, we ran the *MetaEuk* gene predictor (Levy Karin et al., 2020), with the lineage dataset aves_odb10. To identify coordinates for protein coding sequences, we downloaded the reference assembly and annotation of the ruff (*Calidris pugnax*) obtained from NCBI (GCF_001431845.1) and conducted a lift‐over of the coordinates from the annotation GFF3 file through *Liftoff* v1.6.3 (Shumate & Salzberg, 2021), with options ‐polish and ‐flank 0.1, using *minimap2* v2.17‐r941 (Li, 2018) to align gene sequences.

### 2.5 Whole‐genome re‐sequencing, read mapping and variant calling

We re‐sequenced the genomes of multiple individuals at low coverage using short‐read high‐throughput sequencing. Based on the data we obtained through GPS tracking, we selected 54 adult individuals sourced from local Portuguese breeding populations (11 migrants, 43 residents). Only adults were selected for sequencing because they allowed for a more accurate determination of individual migratory strategy (see Section 3). Genomic DNA was extracted from blood stored in ethanol (using the same salt‐based extraction protocol as mentioned above), and DNA quantity and integrity were assessed using a NanoDrop instrument, Qubit dsDNA BR Assay Kit and through agarose gel visualization. Individual whole‐genome libraries were prepared using the TruSeq DNA PCR‐Free kit (Illumina). Libraries were quantified by qPCR using the KAPA Library Quantification Kit, pooled and sequenced to a coverage of 2.1× using 2 × 150 bp reads in an Illumina instrument at Novogene UK. This produced a total of 992,220,763 short reads. Whole‐genome sequencing data are available in the Sequence Read Archive under BioProject PRJNA713582.

Quality of sequencing reads was evaluated using *FastQC* v0.11.8 (https://www.bioinformatics.babraham.ac.uk/projects/fastqc/). Reads were then mapped to our de novo reference assembly with *BWA‐MEM* (Li, 2013) using default settings. Mapping statistics were calculated using *SAMtools* (Li et al., 2009) and custom scripts. Subsequent analyses were carried out under a genotype likelihood framework using the software *ANGSD* v0.930 (Korneliussen et al., 2014). When lower sequencing coverage precludes accurate genotype calling, genotype likelihood approaches allow for effective estimation of population genetics parameters and have thus been increasingly adopted to study non‐model species (reviewed in Lou et al., 2021). When calculating genotype likelihoods in our analyses, we excluded: (1) triallelic positions (‐skipTriallelic 1); (2) positions with base quality below 30 (‐minQ 30); (3) reads with mapping quality below 30 (‐minMapQ 30); (4) secondary and duplicate reads (‐remove_bads 1); (5) reads with multiple best hits (‐uniqueOnly 1); and (6) reads with one or both of the mates not mapping correctly (‐only_proper_pairs 1).

### 2.6 Population genomics

We started our population genomic analyses by conducting a principal component analysis (PCA) on genotype likelihoods using *PCAngsd* with default parameters (Meisner & Albrechtsen, 2018). This program generated a covariance matrix between all individuals, which was used to estimate principal components and individual loadings with the function *prcomp* from *R* v3.6.3 (R Core Team, 2020). We complemented this approach by calculating individual admixture proportions with *NGSadmix* (Skotte et al., 2013). We ran the analysis for several values of *K* (2–6), imposing a minimum minor allele frequency of 0.05. We also calculated, for each pair of individuals, the relatedness index *r*_*xy*_ (Hedrick & Lacy, 2015) from genotype likelihoods using *ngsRelateV2* (Hanghøj et al., 2019). PCA, admixture and relatedness analyses were run with the full dataset, imposing for each position (in addition to the filters outlined above) a minimum and maximum depth across all samples of, respectively, 10 and 200 (‐setMinDepth 10, ‐setMaxDepth 200) and removing positions that did not have data in at least 15 individuals (‐minInd 15).

Next, we investigated levels and patterns of genetic variation in migrant and resident white storks. For these analyses, we randomly subsampled both groups to 10 individuals (excluding the outlier individuals from the PCA, see Section 3) to avoid introducing biases related to sample size. Using *ANGSD*, we started by generating a maximum likelihood estimate of the unfolded site frequency spectrum and used this to calculate pairwise nucleotide diversity (*π*; Nei, 1987) and Tajima's *D* (Tajima, 1989) in 200 kb non‐overlapping windows. Unbiased nucleotide diversity was obtained by dividing the value obtained for each window by the total number of sites passing filters.

### 2.7 Selective sweep mapping

Population censuses detect a consistent and ongoing shift to residency in white storks from Portugal (Figure 1). If this phenotypic shift is mediated by selection acting on genetic variation, the genomes of resident white storks should exhibit significant deviations from neutrality. To detect signatures of selection in the genomes of residents (*n* = 43), we calculated several statistics that look at distinct but complementary properties of sequence data under selection. Specifically, we used: (1) Tajima's *D* (Tajima, 1989), which detects deviations from neutrality by comparing the number of segregating sites and nucleotide diversity; (2) Fay and Wu's *H* (Fay & Wu, 2000), which identifies recent selective sweeps by analysing the frequency distribution of derived alleles; and (3) the composite likelihood ratio statistic (CLR) from *SweepFinder2* (DeGiorgio et al., 2016), which uses the site frequency spectrum to identify loci affected by recent positive selection.

*ANGSD* was used to calculate *D* and *H* through the *thetaStat* module. Since *H* requires the identification of ancestral and derived alleles, we polarized polymorphic sites using sequence information from the closely related maguari stork (*Ciconia maguari*) obtained from NCBI's SRA database (SRR9946656; Feng et al., 2020). We chose this species from the available *Ciconia* species genome data because its phylogenetic placement and geographic context are likely to minimize issues associated with mapping errors and allele sharing with the white stork (de Sousa et al., 2023). We mapped these Illumina data to our reference genome (using the same approach as our own re‐sequencing dataset; mapping rate: 98.77%, coverage: 21.97×), used *ANGSD* to generate a corrected haploid reference sequence (‐doFasta 3), and finally used this corrected reference as an ancestral sequence when calculating the site allele frequency likelihood. For *SweepFinder2*, since the input file requires allele counts, we first used *ANGSD*'s single‐read sampling method (‐doIBS) to randomly subsample one allele per locus from each of the 43 individuals; these counts were used to generate an input file, and *SweepFinder2* was run under default parameters.

Results of the three tests were combined by calculating the de‐correlated composite of multiple signals (DCMS, Ma et al., 2015), which takes into account the correlational structure of the variables to weigh their relative contributions to the combined score. Prior to this analysis, *D* values were multiplied by −1 so that higher values correspond to higher evidence of a selective sweep. Values of −*D*, *H* and CLR were each first converted into fractional ranks to create uniformly distributed probabilities, to which an inverse‐normal transformation was applied. Normalized scores were transformed into *Z*‐scores, and from these, *p*‐values were calculated assuming a normal distribution of the data. Spearman's rank correlation coefficient was calculated for each pair of variables. Finally, correlation coefficients and *p*‐values were used to calculate the DCMS score for each window (the top 1% outlier windows were considered as the most likely candidates for selection).

Although these approaches are suited to detect loci under selection in the resident white stork population, it is likely that a portion of the putative candidates of selection are not associated with the loss of migration, but rather with other local processes. To further filter out our list of potential candidates, we used *ANGSD* to calculate the fixation index (*F*_ST_) between migrants (*n* = 11) and residents (*n* = 43). To test for significant overlap between outliers of this statistic with those of DCMS, we used *SuperExactTest* (https://network.shinyapps.io/superexacttest; Wang et al., 2015). All genome‐wide statistics were calculated in non‐overlapping 50 kb windows; scaffolds smaller than 1 Mb were discarded, resulting in 22,766 windows that were shared between these analyses.

## 3 RESULTS

### 3.1 Individual consistency in migratory behaviour

**FIGURE 2:**
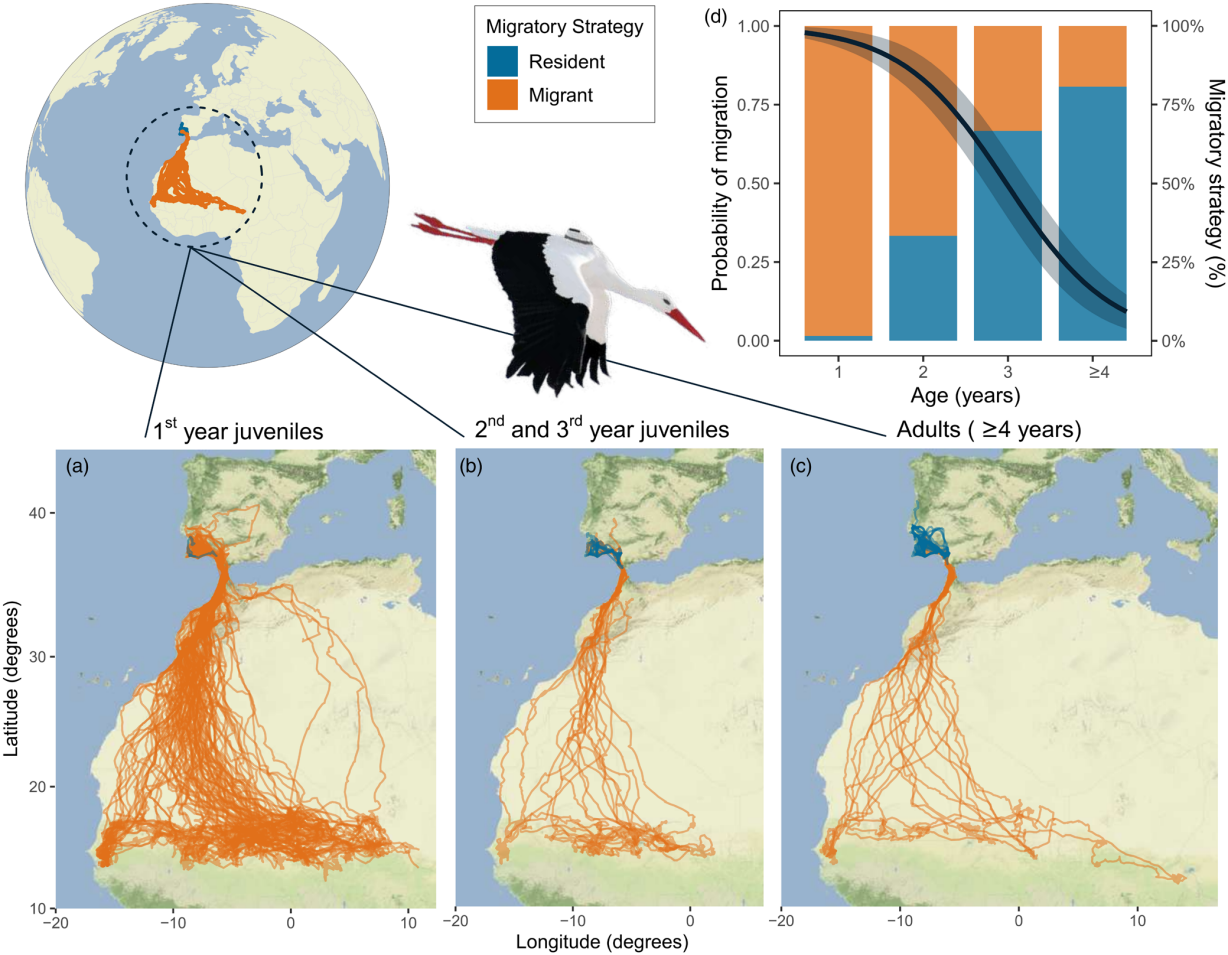
Migratory behaviour of GPS‐tracked white storks from Portugal. Maps show GPS tracks of (a) first‐year juveniles. (b) Second‐ and third‐year juveniles. (c) Adults (≥4 years). Individual routes are coloured according to whether they were classified as migratory (orange, wintering in Africa) or resident, that is, non‐migratory (blue, wintering in Iberia). (d) Probability of migration by age. The regression line (black) was fitted using a binomial generalized linear mixed model testing the effects of age on the probability of migration and 95% confidence intervals (grey area) are shown. Columns reflect the percentage of migratory and resident individuals in each age class (*n* = 133, 24, 12 and 84 individuals with 1, 2, 3 and ≥4 years, respectively). 98% of all first‐year juveniles migrated to Africa, but in their second and third years, the percentage of migratory individuals decreased to 67% and 33%, respectively. Among adults, only 19% of all tracked individuals crossed the Strait of Gibraltar. Three adult individuals that changed migratory strategy between consecutive years were not included.

**FIGURE 3:**
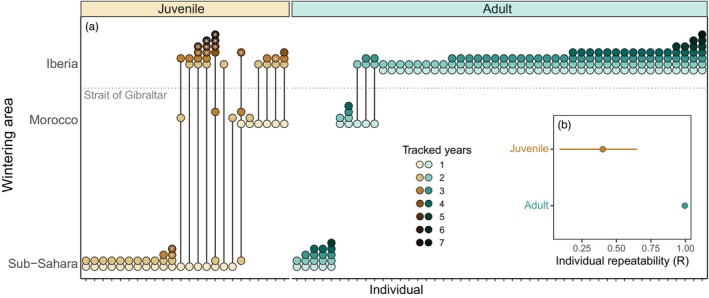
Consistency of white stork migratory behaviour across years. (a) Wintering areas of storks tagged as first‐year juveniles (brown, *n* = 24) and adults (blue, *n* = 48) tracked for multiple years. *X*‐axis represent different individuals and dots represent annual wintering areas, coloured by the number of tracked years (lighter dots show younger birds). Lines indicate shifts in migratory behaviour in consecutive years. Horizontal dashed grey line represents the Strait of Gibraltar: Dots above and below the line show resident (wintering in Iberia) and migratory (wintering in Africa) individuals. Asterisks represent the year in which the juvenile became a breeding adult. (b) Individual repeatability (*R*) in the choice of wintering grounds (minimum latitude reached in October) by first‐year juvenile and adult (>4 years) white storks tracked in multiple years. While adults exhibited high intra‐individual repeatability (*r* = 0.996, SE = 0.001, *p* < 0.0001), juveniles (excluding the breeding years) showed low repeatability (*r* = 0.401, SE = 0.14, *p* = 0.005) in the choice of the wintering region.

GPS tracking of 80 adults (≥4 years old) and 133 juveniles tagged as fledglings revealed extensive variation in the migratory behaviour of white storks (Figure 2a–c) but a gradual decrease in the probability of migration with age (GLMM: −12.8 ± 2.6, *n* = 252, *p* < 0.001; Figure 2d). Despite the considerable inter‐individual variability in the migratory patterns of adult storks (Figure 2c), individuals tracked from 2 up to 7 consecutive years (*n* = 48) exhibited remarkable consistency in their migratory strategy over multiple years, revealing high intra‐individual repeatability in the choice of the wintering latitude (*r* = 0.996, SE = 0.001, *p* < 0.0001, *n* = 161 bird‐year comparisons, Figure 3). Only on three occasions did adult storks change migratory decisions (2% of all birds); one bird in 2017 and two birds in 2021 remained in Iberia after having migrated to Morocco in the previous year (Figure 3a). Overall, among tracked adults, only 19% migrated to Africa (wintering in Morocco or Sub‐Saharan Africa) while 79% of the individuals remained in Iberia (Portugal and Spain), thus corroborating the national survey results (Figure 1c).

Contrarily to adults, juvenile white storks tracked until reaching sexual maturity (i.e. tracked for >1 and up to 3–4 years, *n* = 24) were not consistent in the choice of their wintering grounds in consecutive years (*r* = 0.401, SE = 0.14, *p* = 0.005, *n* = 59 bird‐year comparisons, Figure 3). Among first‐year juveniles, 98% crossed the Strait of Gibraltar towards their African wintering grounds, with 13% travelling shorter distances to winter in Morocco and 87% wintering further in Sub‐Saharan Africa (Figures 2b and 3a). Juvenile storks either decreased or maintained migratory distance (wintering at northern or similar latitudes) as they aged, with only one exception, a bird that wintered in Morocco and in the following year travelled to the Sahel, Figure 3a. Among young storks, the proportion of migrants decreased to 67% and 33% in their second and third year of life, respectively. Moreover, in their third year, only 17% carried out long‐distance movements to reach Sub‐Saharan Africa (Figure 3a). Finally, all first‐year juveniles tracked until the age of first breeding became consistent in migratory strategy as adults, wintering in Iberia or in Africa (*n* = 5 and *n* = 1, respectively, Figure 3a). These results show that the change in migratory behaviour in this long‐lived species is associated with the immature stage (2 to 3–4 years), with the ultimate migratory strategy likely being acquired in the transition to adulthood, and thereupon predominantly maintained as storks establish themselves as breeders.

### 3.2 Population genomics of migrant and resident white storks

To assess the role of genetic variation in white stork migration, we started by assembling a de novo reference genome of the white stork using linked‐read sequencing (Weisenfeld et al., 2017). This resulted in a 1.26‐Gb reference sequence, comprised of 6992 scaffolds (Table S2). A *BUSCO* search for highly conserved single‐copy orthologues using an avian database revealed our genome to be highly complete: of a total of 8.338 genes tested, 8.091 (97.1%) were present in the assembly. A lift‐over of protein‐coding gene sequences from the ruff genome annotation identified the coordinates for 17,535 genes (95.6% of the total 18,342 annotated ruff genes).

**FIGURE 4:**
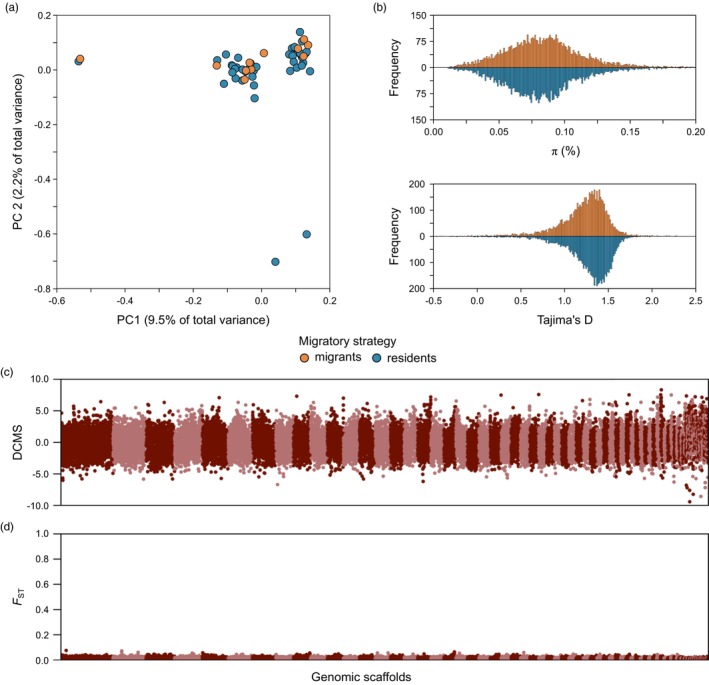
Population genomics of migrant and resident adult white storks. (a) Principal component analysis of genotype likelihoods. The percentage of variance explained by each component is given in parentheses. Individuals are coloured according to their migratory strategy. (b) Frequency distribution of genome‐wide values of nucleotide diversity (*π*) and Tajima's *D* of migrant and resident white storks, calculated in non‐overlapping 200 kb windows. For *π*, a small number of windows above 0.2 were omitted to improve visualization. (c) Signatures of selection on the genome of resident white storks, measured by the de‐correlated composite of multiple signals (DCMS), which summarizes patterns of Tajima's *D*, Fay and Wu's *H* and *SweepFinder2*'s composite likelihood ratio. (d) Genetic differentiation between migrant and resident white storks, measured by the fixation index (*F*_ST_). In panels (c) and (d), the statistics were calculated in non‐overlapping 50 kb windows across the genome; colours indicate alternating genomic scaffolds. Despite differences in migratory strategy between individuals, the genomes of migrant and resident white storks were not differentiated and have similar levels and patterns of nucleotide variation.

Following de novo assembly, we re‐sequenced the genomes of 54 adult birds at 2.1× coverage (migrants and residents, Table S3) and investigated patterns of genomic variation in a sample of migrant and resident white storks. A principal component analysis (PCA, Figure 4a) indicated the presence of two clusters of samples along the first principal component but, most importantly, showed no genetic structure between samples according to their migratory behaviour in either PC1 (Mann–Whitney *U*‐test, *p* = 0.968) or PC2 (Mann–Whitney *U*‐test, *p* = 0.211). An admixture analysis indicates the same pattern, with migrant and resident individuals sharing ancestry to the same clusters along several values of *K* (Figure S4). The low overall genetic differentiation occurred despite no evidence of substantial relatedness between individuals in our dataset. Of a total of 1431 pairwise comparisons between samples, only 10 had an *r*_*xy*_ index above 0.03125 (1/32), corresponding to a relatedness higher than second cousins (average ± standard deviation; *r*_*xy*_ = 0.002 ± 0.013). Concordantly with observations that migrant and resident individuals share the same ancestry and belong to a single interbreeding population, the average genome‐wide nucleotide diversity (*π*) and a measure of the allele frequency spectrum of mutations (Tajima's *D*) are similar between groups (average ± standard deviation; *π*_migrants_ = 0.085 ± 0.045; *π*_residents_ = 0.083 ± 0.041, *D*_migrants_ = 1.219 ± 0.276; *D*_residents_ = 1.279 ± 0.262, Figure 4b). Overall, our results indicate that the recent abrupt shift towards the loss of migratory behaviour in white storks, which leads to large inter‐individual differences in movement patterns, is not associated with differentiated populations within its Iberian breeding grounds.

To examine evidence of recent selection associated with loss of migratory behaviour, we scanned the genome of resident storks for signatures of selective sweeps using statistics that highlight different properties of genetic variation (Tajima's *D*, Fay and Wu's *H* and *SweepFinder2*'s CLR, Figure S5). As expected, overall correlation coefficients between the three tests had a wide range of variability (*ρD‐H* = 0.400; *ρD‐*CLR = 0.199; *ρH‐*CLR = −0.109). These should, however, converge in genomic regions under selection, so we summarized this information with the composite statistic DCMS (Figure 4c). The top 1% of the empirical distribution of DCMS (228 non‐overlapping 50 kb windows) overlapped the full open‐reading frame of 223 protein coding genes. None of these windows was an outlier in all the three statistics independently, and the majority represented isolated windows (197 independent regions, once adjacent windows were merged). The top outlier region (scaffold50: 1,200,000–1,450,000), contained four genes, *EPHB6* (ephrin type‐B receptor 6) and three T‐cell receptor β chain genes. Other large outlier regions, containing three or more adjacent windows, were located on scaffold10 (6,550,000–6,700,000; 2 genes), scaffold17 (23,550,000–23,750,000; 14 genes), scaffold19 (19,700,000–19,850,000; 3 genes) and scaffold49 (1,450,000–1,600,000; 1 gene).

While these genomic regions may represent evidence for selection acting on the resident stork population, it is possible that they reflect selection on traits not associated with migration. To test this, we explicitly calculated genome‐wide differentiation (*F*_ST_) between migrant and resident storks. Strikingly, we failed to find substantial differences between individuals of the two groups at any locus across the genome (Figure 4d). The highest *F*_ST_ values were also scattered throughout the genome (182 independent regions, once adjacent windows were merged) and, more importantly, were of very low magnitude. To illustrate this, the top window had an *F*_ST_ of 0.076, and only 15 windows (0.07% of the total number) had an *F*_ST_ above 0.05. The average *F*_ST_ in the top 1% DCMS windows (*F*_ST_ = 0.015) was also similar to the genome‐wide average (*F*_ST_ = 0.014). A comparison of the top DCMS outliers with the top *F*_ST_ outliers indicated a general lack of concordance (Figure S6), since only four genomic windows were simultaneously 1% outliers in both statistics, an overlap that was not higher than expected by chance (*p* = 0.196). Of these four windows, three were placed on scaffold3 (no gene was located 100 kb either side of these windows) and a window on scaffold40 (overlapping genes *HAO2* and *3BHSD*); patterns of *F*_ST_ between the two groups in these genomic regions do not conclusively suggest an association (Figure S6).

This likely lack of a genetic association in our study does not preclude a scenario of selection acting on an unassembled region of the genome (unlikely given our genome is highly complete), or that the shift could be associated with widespread polygenic selection characterized by very small changes in allele frequency (which would require a very large sample size to detect). However, taken together with our tracking data, these results are consistent with a scenario in which developmental effects are the major mechanisms explaining the rapid loss of migration in white storks, albeit with potential minor contributions from phenotypic flexibility or selection on genetic variation. These results were further supported by a population viability analysis (PVA; see Supplementary text, Figure S9, Tables S4 and S5; Section 4) simulating a shift in the population towards residency without considering adult phenotypic plasticity.

## 4 DISCUSSION

Unveiling if, and how, natural populations respond to ongoing human‐driven environmental changes is a topic of central importance in evolutionary ecology and conservation. Given the potential for animal migration to alter ecological networks worldwide (Bauer & Hoye, 2014), understanding the roles that selection and plasticity play in shaping migratory strategies will be key to comprehend how this life‐history trait can change in response to environmental changes. Our results show that rapid and drastic population‐level changes in avian migratory behaviour, including the loss of migration itself, can arise through generational shifts, with young recruits as the agents of such changes through developmental plasticity. In white storks, the documented shortening of migratory distances, and even the loss of migratory behaviour (Archaux et al., 2004; Catry et al., 2017; Cheng et al., 2019; Flack et al., 2016; Rotics et al., 2017), is likely occurring through inter‐generational processes driven by the recruitment of non‐migratory young storks into the population. This could represent an effective mechanism for rapid acclimation to anthropogenic environmental change.

Individual long‐term tracking of adult white storks revealed high within‐individual consistency in migratory strategy (either migratory or non‐migratory), thus not supporting recent findings in other bird species reporting phenotypic flexibility as the mechanism driving population‐level changes in avian migratory behaviour (Conklin et al., 2021; Fraser et al., 2019). Between 2016 and 2022, only three adult storks shifted migratory strategy (3 out of 113 potential transitions), from short‐distance migration to Morocco to residency in Iberia. This observed adult flexibility (0.4% per year) would not be enough to explain the magnitude of observed changes in the migratory behaviour of the Portuguese stork population in the last 25 years, where 50%–64% of the population became non‐migratory (Figure 1c). In addition, our tracking data capture a period of significant shifts in migratory behaviour: over just 6 years (2014–2020), we estimate that the proportion of residents increased by 8%–23%. Considering the exceptionally high level of within‐individual consistency in migratory behaviour among adults (*R* = 1), it is highly unlikely that these changes in the population were driven by adult phenotypic plasticity. One can argue that the high consistency in migratory strategy among resident adults does not derive from an inflexible phenotype, but instead from the favourable and stable environmental conditions in Iberia during the non‐breeding season and due to high food availability in landfills. This study does not demonstrate that adult behaviour is irreversible. Yet, if adults had a flexible phenotype, we would expect to observe some degree of plasticity in migrants, particularly those travelling long distances. Within our population, long‐distance migrants incur in higher overwintering energetic costs compared to residents (Soriano‐Redondo et al., 2023) and likely experience inter‐annual variability in wintering conditions in the Sahel (Zwarts et al., 2009). Moreover, and similarly to residents, migratory storks rely heavily on landfills during the breeding season (Soriano‐Redondo et al., 2021; Supplementary text, Figure S8), suggesting they could access and secure these abundant and predictable resources also during the non‐breeding season. However, the increased food resources and suitable environmental conditions in Iberia have not, so far, disrupted the migration of adult storks.

Contrarily to adults, most tracked juveniles migrated in the first year and then shifted towards residency. This shift occurred throughout their development, before they reached sexual maturity (3–4 years), suggesting that a population can transition from predominantly migratory to mostly resident through inter‐generational shifts in the frequency of non‐migratory recruits. This is supported by a population viability analysis showing that a comparable phenotypic shift at the population level can be attained by a conversion rate of just 10% of migratory juveniles to resident adults, without considering adult phenotypic plasticity or reversibility of adult migratory behaviour (Supplementary text, Figure S9, Tables S4 and S5).

As a social species, we would expect that most first‐year storks would remain in Iberia, alongside non‐migratory adults. Yet, the species' innate migratory programme likely drives all first‐year juveniles to complete an initial migration to Africa (Chernetsov et al., 2004; Mayr, 1952), possibly guided by immature or adult migratory individuals from their own or other populations to successfully reach their wintering areas. In their second and third years, environmental, physiological, individual experience or social cues could decrease the propensity to migrate and override the initially expressed migration program, leading to highly consistent adult phenotypes (Åkesson & Helm, 2020; Gill et al., 2019; Méndez et al., 2021). Indeed, while 98% of first‐year juveniles migrated to Africa, the proportion of migratory individuals decreased to 67% and 33% in their second and third years. Moreover, first‐year juveniles tracked beyond the age of first breeding maintained their migratory strategy throughout adulthood (five juveniles became residents and one remained migrant, wintering in the Sahel), adding to the evidence that migratory strategy may not be fully behaviourally flexible, stabilising at a certain stage of individual development.

Although, for many species, the propensity to migrate long distances has a strong genetic component (Liedvogel, 2019) that could be under selection due to environmental change, we did not find conclusive support for the hypothesis of an evolutionary response to selection driving the loss of migration in the white stork. It should, however, be noted that our limited sample size may preclude a definitive answer on the role of adaptive evolution if it is associated with soft sweeps on multiple loci of small effect (Hermisson & Pennings, 2017). In any case, the lack of a clear signal across several independent statistics is indicative that a recent sweep based on a large effect locus, as found in previous genomic studies, is unlikely. While the innate migratory programme presents an opportunity for selection to drive changes in migratory strategy, plasticity should provide a faster mechanism for adaptation. An association between changes in migratory strategy and large‐effect genetic variation is thus more likely to be found in smaller birds (nocturnal solitary migrants) (Delmore et al., 2016, 2020; Lundberg et al., 2017; Sanchez‐Donoso et al., 2022; Toews et al., 2019), which are typically short‐lived and thus less likely to modulate migration based on ontogenetic effects or experience (Pulido, 2007).

The observed shortening of migration distance and suppression of migratory behaviour in white storks has been associated with an increased year‐round food availability from landfills (Catry et al., 2017; Cheng et al., 2019; Flack et al., 2016). Recent studies showed that foraging on landfill waste is a time‐ and energy‐saving strategy that enables storks to reduce their movement and foraging efforts (Soriano‐Redondo et al., 2021), thus facilitating their survival through the winter season. Indeed, overwintering in North Africa (Morocco) and Europe, where landfills and rubbish dumps provide a high abundance of food, has been shown to enhance white storks' juvenile survival (Cheng et al., 2019; Rotics et al., 2017), likely contributing towards the manifestation of non‐migratory behaviour. For immatures, the decision to stay in Iberia and no longer migrate to their sub‐Saharan wintering grounds could also be strengthened by social learning (Byholm et al., 2022; Mueller et al., 2013; Teitelbaum et al., 2016), in which young storks acquire information from the increasing number of experienced conspecifics overwintering on these food waste disposal sites. Despite the evident benefits, foraging on landfill waste is also likely to exacerbate intraspecific competition (Martins et al., 2024; Soriano‐Redondo et al., 2021). Thus, social interactions, mediated by each individual's foraging proficiency and experience, could function to promote or suppress migration of dominant and outcompeted individuals, respectively (Campioni et al., 2020; Grecian et al., 2018). Finally, migratory propensity could also be altered during maturation through carry‐over effects of differential migratory performance. Increased flight costs have been shown to be an important proximate cause of storks' juvenile mortality during migration (Rotics et al., 2017) and poor individual performance during first migrations could suppress migratory behaviour in following years. Regardless of the drivers behind shifts in migratory behaviour during juvenile development, disproportionate differences in survival rates of migratory and non‐migratory recruits could, over generations (i.e. through selective mortality), accelerate the white stork population turnover towards residency. In the short term, if environmental conditions continue to favour non‐migratory individuals, the white stork population is likely to change towards full residency. Yet, future waste reduction initiatives planned by the European Union (Soriano‐Redondo et al., 2021) might revert this tendency, operating through food shortages and density‐dependent processes (e.g. increase competition) during the non‐breeding period. In such a scenario, we hypothesize that storks will either (i) remain resident if they find alternative food sources in the breeding grounds (e.g. crayfish in rice fields) or (ii) adapt to future environmental changes by resuming migration through developmental plasticity, with migratory juveniles replacing resident adults over generations.

Advances in tracking technology now allow us to monitor animals with unprecedented detail along their annual cycles and entire lifespans (e.g. Börger et al., 2020). Concurrently, ever‐improving genome sequencing technologies have greatly expanded our knowledge of trait evolution in natural systems. Merging these approaches can revolutionize our understanding of animal movement and migratory behaviour by helping us connect phenotypic responses to their underlying evolutionary and developmental processes. Our study expands on earlier findings on the evolution and development of bird migratory behaviour in three key aspects. First, we offer additional evidence that these developmental processes can lead not only to phenological and range changes in migratory movements (Gill et al., 2019; Verhoeven et al., 2018, 2021) but also the undertaking of long‐distance migration itself. Second, we present evidence that genetic variation, in particular in large‐effect loci, is likely not associated with this shift. Third, we employed a large‐scale longitudinal study throughout the earlier years of white storks, pinpointing the developmental interval during which dramatic changes in behaviour take place. By allowing organisms to develop phenotypes adjusted to the conditions that adults will experience, developmental plasticity can provide, through generational shifts, a fast mechanism for long‐lived species to adapt to novel ecological opportunities within the lifespan of individuals and the topic is receiving increased attention (Åkesson & Helm, 2020; Gill et al., 2019). Future research should thus focus on identifying the specific developmental mechanisms that drive migratory traits during ontogeny (e.g. flight efficiency, migratory performance, access to food resources at landfills) to further increase our understanding about species adaptation to environmental change and the associated implications for their conservation.

## AUTHOR CONTRIBUTIONS

I.C., A.M.A.F., F.M. and M.A. coordinated and performed the collection of ecological and tracking data from white storks. M.C., S.A. and C.I.M. coordinated and performed the collection of genetic data. P.A., M.C. and C.I.M. performed genetic data analyses. I.C., M.A. and A.M.A.F. performed ecological and tracking data analyses. P.A. and I.C. wrote an initial version of the manuscript, which had input from all other authors.

## FUNDING INFORMATION

This work was financed by the FEDER Funds through the Operational Competitiveness Factors Program—COMPETE and by National Funds through FCT (Fundação para a Ciência e Tecnologia) within the scope of the project Birds on the move ‘POCI‐01‐0145‐FEDER‐028176’, and POCI‐01‐0145‐FEDER‐006821, with support from the REN Biodiversity Chair and by the Natural Environment Research Council (NERC), via the EnvEast DTP, and NERC and Engineering and Physical Sciences Research Council (EPSRC), via the NEXUSS CDT Training in the Smart and Autonomous Observation of the Environment (NE/R012156/1). Funding for the development of the GPS tracking devices was provided by NERC (NE/K006312), Norwich Research Park Translational Fund, University of East Anglia Innovation Funds and Earth and Life Systems Alliance funds. P.A. was supported by FCT through a research contract in the scope of project PTDC/BIA‐EVL/28621/2017 and research contract 2020.01405.CEECIND/CP1601/CT0011. M.A. was supported by NERC, via the NEXUSS CDT (NE/R012156/1). C.I.M. was supported by FCT through a research grant (SFRH/BD/147030/2019) in the scope of the Biodiversity, Genetics, and Evolution (BIODIV) PhD program. M.C. was supported by FCT through POPH‐QREN funds from the European Social Fund and Portuguese MCTES (CEECINST/00014/2018/CP1512/CT0002). I.C. and F.M. were supported by FCT (contract numbers 2021.03224.CEECIND and IF/01053/2015, respectively). Work supported by National Funds through FCT in the scope of the project LA/P/0048/2020.

## CONFLICT OF INTEREST STATEMENT

The authors declare no conflicts of interest.

## Supporting information


**Figure S1:** Number and distribution of wintering white storks in Portugal during the last 25 years.
**Figure S2:** Number and origin (PT—Portuguese, Non‐PT—other countries) of white storks resighted at four Portuguese landfill sites from September to December 2019–2020 (mean number of storks in the four landfills = 5100, 4850, 5075 and 3650 in September, October, November, and December, respectively).
**Figure S3:** Histogram of the yearly maximum distances to the nest (in km) of storks wintering in Iberia (pink) and in Morocco (blue).
**Figure S4:** Individual admixture proportions for migratory (*n* = 11) and resident (*n* = 43) white storks, calculated using *NGSadmix*.
**Figure S5:** Manhattan plots with genome‐wide scans for signatures of selection in resident white storks.
**Figure S6:** Comparison between values of genetic differentiation (fixation index, *F*_ST_) and the decorrelated composite of multiple signals (DCMS, summarizing patterns of Tajima's *D*, Fay and Wu's *H*, and *SweepFinder2*'s composite likelihood ratio), for 22,766 genomic windows of 50 kb (non‐overlapping).
**Figure S7:** Genetic differentiation (fixation index, *F*_ST_) between migrant and non‐migrant white storks, at the three genomic regions that were 1% outliers in the *F*_ST_ and DCMS statistics (corresponding to the four windows in Figure S5).
**Figure S8:** Proportion of landfill days during the breeding season (March, April and May) for migrant and resident white storks.
**Figure S9:** *Vortex10* simulations of white stork population trajectories obtained for 26 years from 1994 to 2020.
**Table S1:** Number of adult and juvenile white stork (*Ciconia ciconia*) GPS‐tracked between 2 and 7 years.
**Table S2:** Summary statistics for the *de novo* genome assembly for the white stork *Ciconia ciconia* (Ccic_1.0).
**Table S3:** Summary statistics of the whole‐genome re‐sequencing dataset.
**Table S4:** *Vortex10* parameters that were used to model the changes in the number of resident and migratory white storks in Portugal.
**Table S5:** Population demography parameters from *Vortex10* comparing different migratory strategies for the Portuguese white stork population.

## Data Availability

Chromium sequencing data for reference genome assembly and whole‐genome re‐sequencing data are available in the Sequence Read Archive (www.ncbi.nlm.nih.gov/sra) under BioProject PRJNA713582.
