# Supplementary material for: Mechanisms underlying the loss of migratory behaviour in a long‐lived bird
Source: J Anim Ecol. 2025 Apr 2;94(5):1061–75. doi: 10.1111/1365-2656.70035 (PMC12056346; doi:10.1111/1365-2656.70035)
Supplement: Supplementary file 1 — Figure S1: Number and distribution of wintering white storks in Portugal during the last 25 years. Figure S2: Number and origin (PT—Portuguese, Non‐PT—other countries) of white storks resighted at four Portuguese landfill sites from September to December 2019–2020 (mean number of storks in the four landfills = 5100, 4850, 5075 and 3650 in September, October, November, and December, respectively). Figure S3: Histogram of the yearly maximum distances to the nest (in km) of storks wintering in Iberia (pink) and in Morocco (blue). Figure S4: Individual admixture proportions for migratory (n = 11) and resident (n = 43) white storks, calculated using NGSadmix. Figure S5: Manhattan plots with genome‐wide scans for signatures of selection in resident white storks. Figure S6: Comparison between values of genetic differentiation (fixation index, F ST) and the decorrelated composite of multiple signals (DCMS, summarizing patterns of Tajima's D, Fay and Wu's H, and SweepFinder2's composite likelihood ratio), for 22,766 genomic windows of 50 kb (non‐overlapping). Figure S7: Genetic differentiation (fixation index, F ST) between migrant and non‐migrant white storks, at the three genomic regions that were 1% outliers in the F ST and DCMS statistics (corresponding to the four windows in Figure S5). Figure S8: Proportion of landfill days during the breeding season (March, April and May) for migrant and resident white storks. Figure S9: Vortex10 simulations of white stork population trajectories obtained for 26 years from 1994 to 2020. Table S1: Number of adult and juvenile white stork (Ciconia ciconia) GPS‐tracked between 2 and 7 years. Table S2: Summary statistics for the de novo genome assembly for the white stork Ciconia ciconia (Ccic_1.0). Table S3: Summary statistics of the whole‐genome re‐sequencing dataset. Table S4: Vortex10 parameters that were used to model the changes in the number of resident and migratory white storks in Portugal. Table S5: Population demography paramet [file JANE-94-1061-s001.pdf]

Mechanisms underlying the loss of migratory behaviour in a long-lived bird

SUPPLEMENTARY TEXT

Non-breeding surveys and estimates of resident white storks in Portugal

The number of resident white storks was assessed from the number of individuals counted during the non-breeding surveys, performed from mid-September to early October. This period was chosen because most migratory individuals cross the Strait of Gibraltar towards their African wintering grounds between July and early September (Fernández-Cruz et al., 2005; Soriano-Redondo et al., 2020); indeed, among our tracked adult and juvenile storks, autumn migration started between 7th of July and 4th September (median date = 5th August, SD = 17 days, *n* = 75; Acácio et al. 2022). The pre-nuptial return migration to the breeding areas starts in November (Fundación MIGRES, pers. comm.) and none of our GPS-tracked storks arrived in Portugal before November. However, counts performed during this period could overestimate the number of resident storks due to the inclusion of storks of non-Portuguese origin that overwinter in Portugal. Nonetheless, most of the storks from the Central and Northern European populations migrating through or spending the winter in Iberia, seem to select further eastern routes/wintering areas, and the ones traveling to Portugal, seem to arrive mostly from mid-October onwards. Indeed, publicly available tracking data on Movebank studies with available visualization of tracks, show that only 9 out of 342 white storks migrating through/to Iberia visited Portugal (number of storks tagged in Austria = 5, France = 29, Germany = 236 and Spain = 72). Careful observation of individual tracks and data available on the Movebank Data Repository shows that only 1 out of the 9 storks visiting Portugal was present during the survey period (15 Sep-15 Oct), thus representing less than 1% (0.003%) of all tracked individuals.

Resights of ringed white storks at Portuguese landfill sites during monthly counts in 2019 and 2020 also support the hypothesis that most foreign individuals (from other European countries) arrive from late October (Figure S2). Overall, the inclusion of non-Portuguese storks in the censuses of resident storks should be negligible and likely compensated by the absence of Portuguese resident storks wintering in southern Spain (observed from GPS tracked individuals).

Movebank studies: Cicognes de Loire-Atlantic; Ciconia ciconia Sudewiesen; Cicognes de Saintonge; HUJ MPIAB White Stork E-obs; HUJ MPIAB White Stork GSM 2013; HUJ MPIAB White Stork GSM E-obs; Life Track White Stork Bavaria; Life Track White Stork Catalonia; Life Track White Stork Loburg 2022; Life Track White Stork Oberschwaben; Life Track White Stork Rheinland-Pfalz; Life Track White Stork Sarralbe; Life Track White Stork Spain Donana; Life Track White Stork SW Germany; Life Track White Stork SW Germany Care Centre Release; Life Track White Stork SW Germany CASCB; Life Track White Stork Vorarlberg; MPIAB Argos white stork tracking (1991-2022); White Stork Affenberg releases MPIAB; White Stork Loburg 2014.

Movebank data repository accessions: doi:10.5441/001/1.v1cs4nn0; doi:10.5441/001/1.c42j3js7; doi:10.5441/001/1.4192t2j4; doi:10.5441/001/1.ck04mn78; doi:10.5441/001/1.71r7pp6q

Population viability analysis

White stork numbers increased in Portugal in the last few decades (Catry et al., 2017) following a period of population declines observed in the first half of the 20th century (BirdLife International, 2016). In Portugal, census data show the breeding population increased 350%, from 3302 breeding pairs in 1994 to 11691 breeding pairs in 2014. During this period, the proportion of resident storks increased from 18% to 61.7%. A subsequent winter census in 2020 counted 19,282 white storks wintering in Portugal, corresponding to 67.6-82.5% of the breeding population.

We used a population viability analysis (PVA), incorporating biological and environmental variables, to explain the observed changes in the number of migrant and resident white storks in the last decades, and compared two scenarios: (1) populational shift towards residency explained solely by differences in demographic parameters between resident and migrant storks, with no individual changes in migratory behaviour; (2) populational shift towards residency explained by the loss of migratory behaviour during ontogeny, considering the conversion of juvenile migrants to residents, followed by consistency in migratory strategy in the adult life stages, as hypothesized in this study.

Methods

We predicted the trajectories of the migrant and resident populations from 1994 to 2020 using the average demographic parameters for our monitored white stork population (authors' unpublished data, Soriano-Redondo et al., 2023). When parameters were not available for the Portuguese white storks, we used the average from Western European white stork populations summarised in Mayall et al. (2023). The demographic parameters used and the two scenarios are summarised in Table S4. The demographic trajectories for both migratory and resident populations were obtained using *Vortex* 10.5.20 (Lacy, 2019). *Vortex10* is an individual-based simulation model in which populations are subjected to a set of deterministic environmental, demographic, and genetic stochastic events (Brook et al., 1999). The rationale for the different parameters that were applied was as follows:

a) **Initial population size**: The initial population size was set to 5,416 migratory and 1,188 resident white storks, as obtained during the first breeding and wintering census in 1994

(Catry et al., 2017). We ran the demographic models for 26 years, from 1994 to 2020, as this was the year when the last census was performed and for which we have demographic data, reducing the influence of uncertainties in parameter estimations.

b) **Dispersal**: We modeled two scenarios. In scenario 1, we investigated if the differences in demographic parameters of the migratory and resident storks could drive the observed population changes (i.e., no individual changes in migratory strategy, hence no dispersal between the two populations). Scenario 2 included 10% of dispersal from the migratory to the resident population, during the storks' 2nd and 3rd years of life, simulating the observed changes in the migratory behaviour of juvenile storks. The current observed rates of dispersal reported in this study are higher, yet these rates are likely to have changed through time, hence 10% was considered an average value over 26 years modelled.

c) **Reproductive system**: White storks are socially monogamous, with high nest fidelity (Barbraud et al., 1999) and moderate levels of extra-pair paternity (Turjeman et al., 2016). Therefore, we described the mating system as long-term monogamous relationships in *Vortex10*. The maximum age of breeding for both males and females was set to 30 (Mayall et al., 2023), with one brood per year. Age of first breeding can vary but generally starts in the 3rd year of life (Barbraud et al., 1999; Hancock et al., 1992; authors' unpublished data); there are some recorded cases of two-year-old storks attempting to reproduce, although rarely successfully (Barbraud et al., 1999).

d) **Reproductive rate**: We assumed that 100% of adult females aged three years and older would attempt to breed with a 10% standard deviation due to environmental variation. Based on the average information obtained from 2016 to 2020, 95% of the females successfully fledged young at a rate of 1.7 (SD=0.45) fledglings per successful nest (authors' unpublished data). The maximum progeny per brood was set to four (authors' unpublished data). There were no differences in reproductive rates between the two populations as reported in Soriano-Redondo et al. (2023).

e) **Mortality rates**: Mortality rates vary significantly with age, both for migrants and resident storks. In the first year of life, most storks migrate, hence mortality is the same for both populations. First-year storks have high mortality rates due to low foraging experience and less efficient flight strategies (Kanyamibwa et al., 1990; Rotics et al., 2016). Collisions with power lines can also contribute heavily to post-fledging mortality (Tobolka, 2014).

Juveniles (ages 0–1) were assigned a mortality rate of 65.1% (SD due to environmental variation = 10) based on a mean value weighted by sample size for the western European stork population (extracted from Mayall et al. 2023). In the second year of life mortality declines, and was set at 22.2% (SD due to environmental variation = 3) for migratory and 17.7% for resident storks based on average values obtained from Mayall et al. (2023). The mortality of migratory birds 3+ was set at 11% (SD due to environmental variation =3) for residents and 9% (SD due to environmental variation =3) for the migratory birds (Soriano-Redondo et al., 2023).

f) **Genetics**: The white stork's long lifespan and slow generation time may result in the presence of deleterious genetic effects which may be hidden. *Vortex10* considers the lethal equivalent (LE) as a unit of deleterious genetic variation that when dispersed amongst a group of individuals could result in the mortality of specific individuals (Kalinowski & Hedrick, 1998). Due to high dispersal and strong population growth rates, all models were considered to have 0 LE. There is evidence to suggest that European white storks did not lose a significant amount of genetic diversity following their twentieth-century decline, suggesting that any negative repercussions from inbreeding depression are unlikely to have hindered the Portuguese populations (Shephard et al., 2013).

g) **Carrying capacity**: The carrying capacity was set at 35,000 which is well above the population sizes observed at present. This value was selected to not limit population growth rates. There were no other management measures included in the simulations.

Results

The demographic parameters used to determine the population trajectories simulated well the increase in white stork numbers observed over the 26 years (Figure S9), the overall white stork population increased to 28,842-31,862 individuals, according to scenarios 1 and 2 respectively (Table S5). Both simulated populations are similar to the current white stork population size (28,500 storks). However, in scenario 1 (no changes in migratory behaviour) the percentage of resident storks at the end of the 26 years, in 2020, (26.9%, Figure S9) is significantly lower than the observed percentage in our studied population (67.6 – 82.5%, Figure 1C in the manuscript), while in Scenario 2 (including 10% dispersal from the migratory to the resident population during

ontogeny), the number of residents increased sharply and the percentage of resident individuals (69.8%, Table S5) is similar to the observed percentage in our studied population.

These simulations show that the ongoing changes in migratory behaviour in the Portuguese white stork population would not be possible without the conversion of migratory to resident storks. This conversion was only observed during ontogeny (2-3 years), as adults were consistent in their migratory strategy. Thus, we provide additional evidence that the observed turnover in the population migratory traits is occurring through generational shifts (with migratory juveniles settling as resident adults in the metapopulation).

SUPPLEMENTARY FIGURES

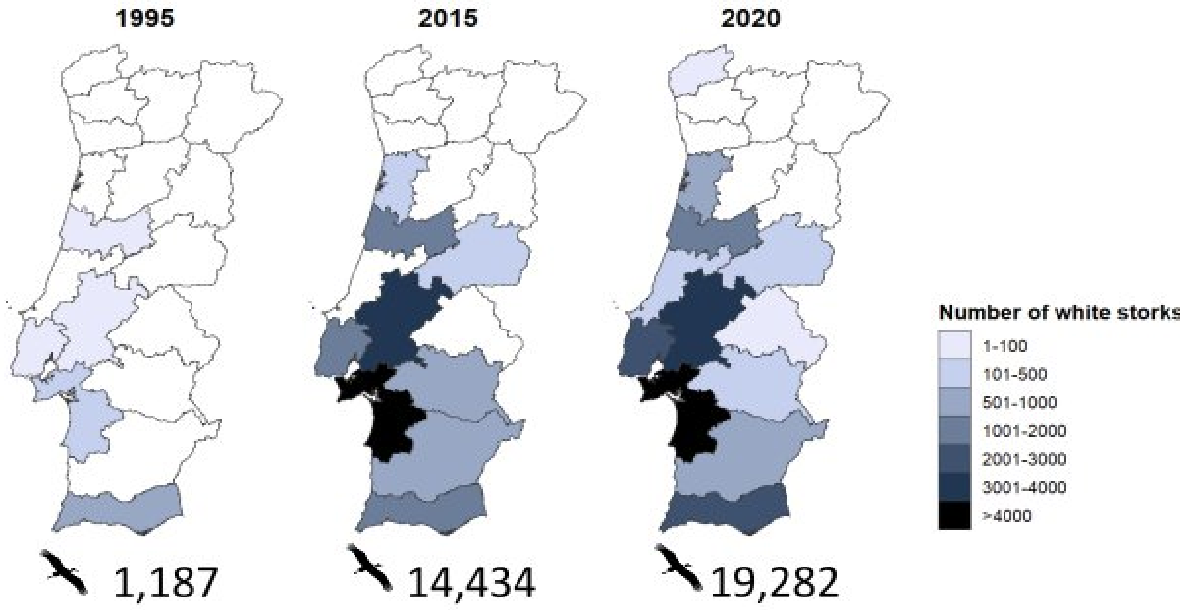

Figure S1. Number and distribution of wintering white storks in Portugal during the last 25 years. The total number of individuals counted during the non-breeding census in 1995, 2015 and 2020 is shown below the respective map.

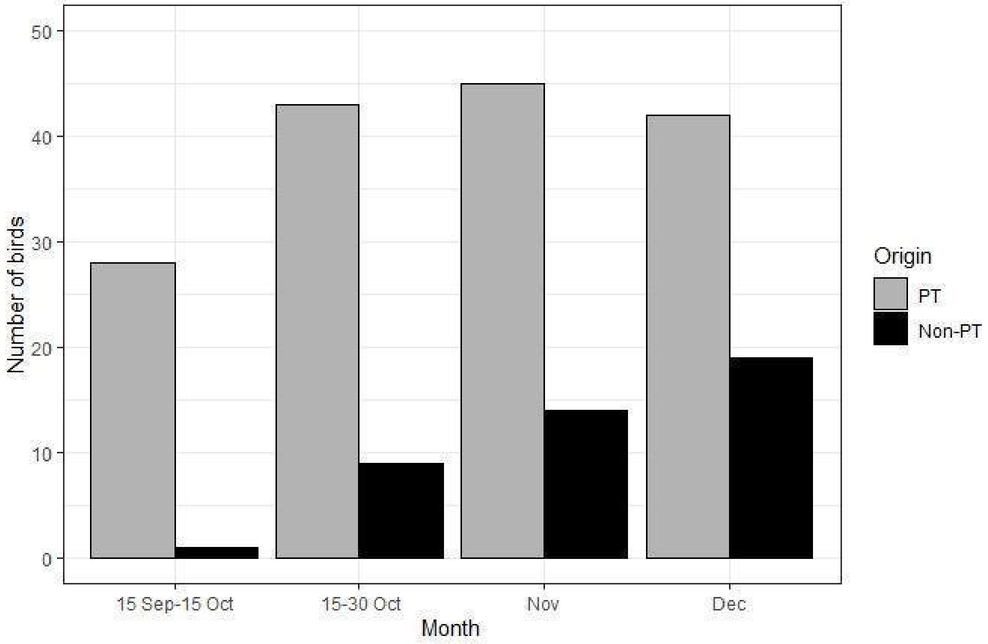

Figure S2. Number and origin (PT - Portuguese, Non-PT - other countries) of white storks resighted at four Portuguese landfill sites from September to December 2019-2020 (mean number of storks in the four landfills = 5100, 4850, 5075 and 3650 in September, October, November, and December, respectively). These data indicate that the overwhelming majority of birds detected during non-breeding surveys (carried out before the 15th of October) were non-migrants of local origin.

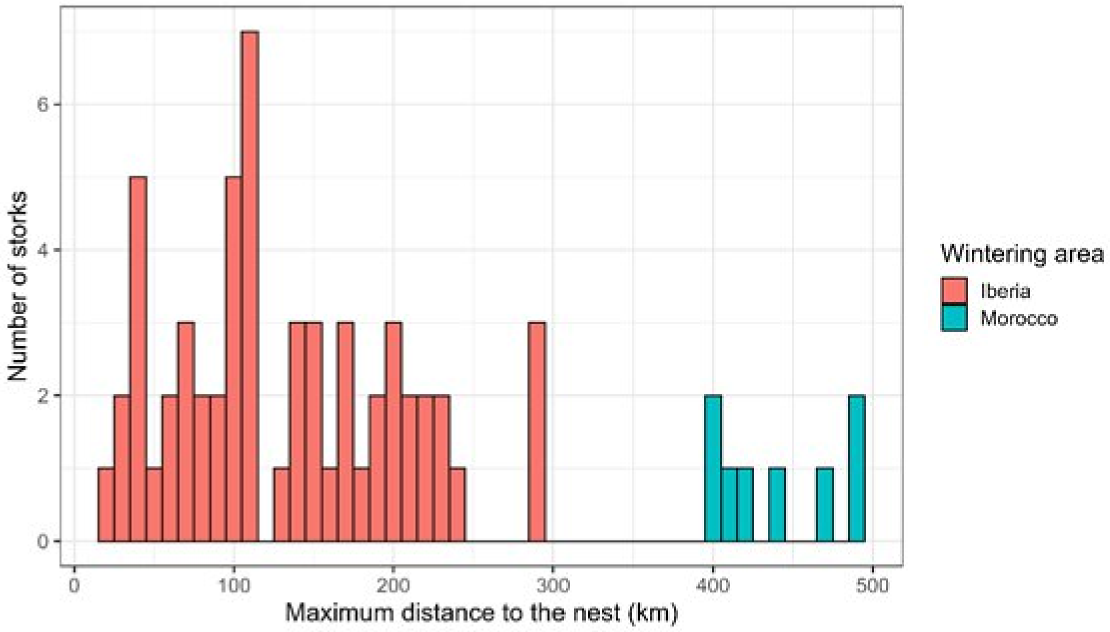

Figure S3. Histogram of the yearly maximum distances to the nest (in km) of storks wintering in Iberia (pink) and in Morocco (blue). To minimize biases, due to following some individuals for multiple years, this graph only shows 1 stork-year per bird. This year was selected as the year the stork travelled the furthest distance away from its nest, excluding all stork-years in which the stork was not tracked at least until October. This graph includes 65 stork-years (between 2016 and 2022), 57 Iberian and 8 Moroccan.

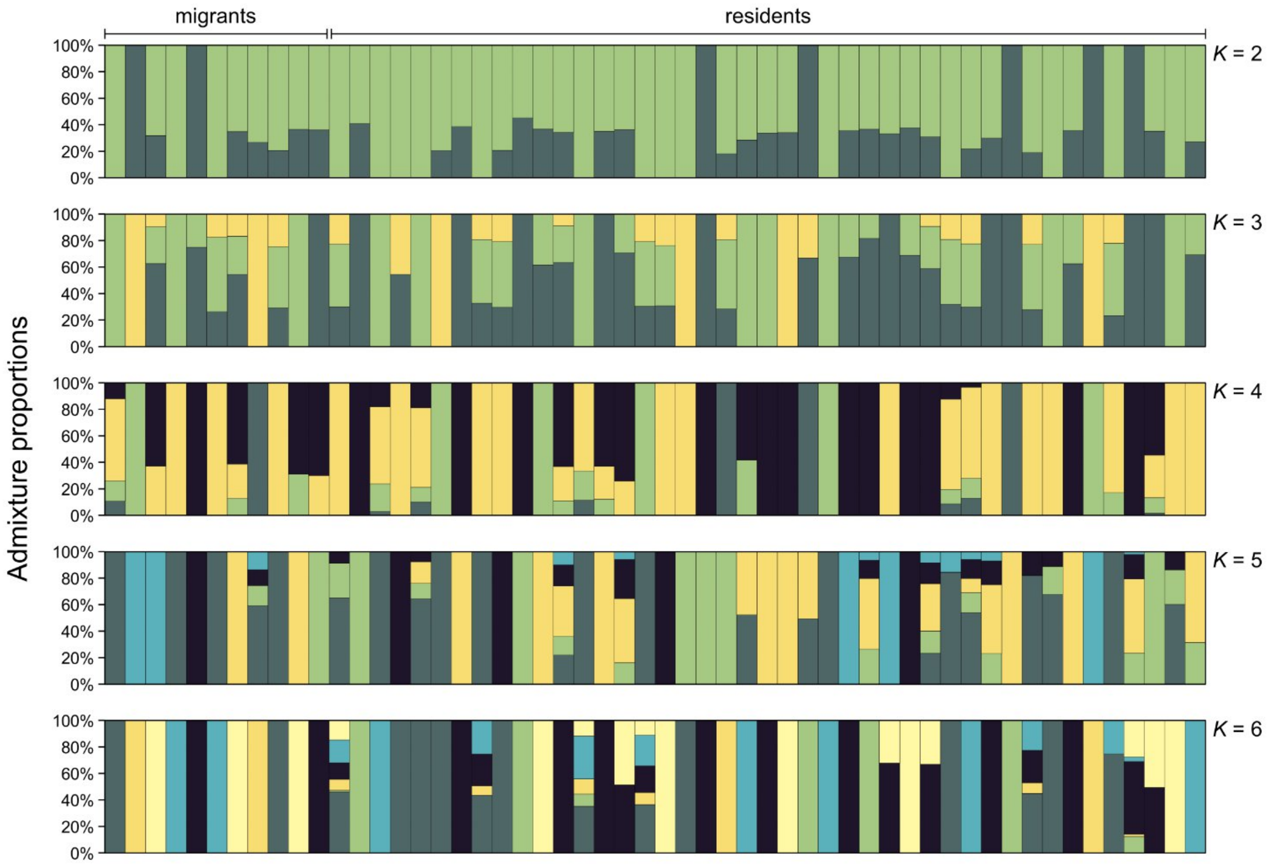

Figure S4. Individual admixture proportions for migratory (n = 11) and resident (n = 43) white storks, calculated using *NGSadmix*. Results for several values of K are shown ($K = 2$ to $K = 6$).

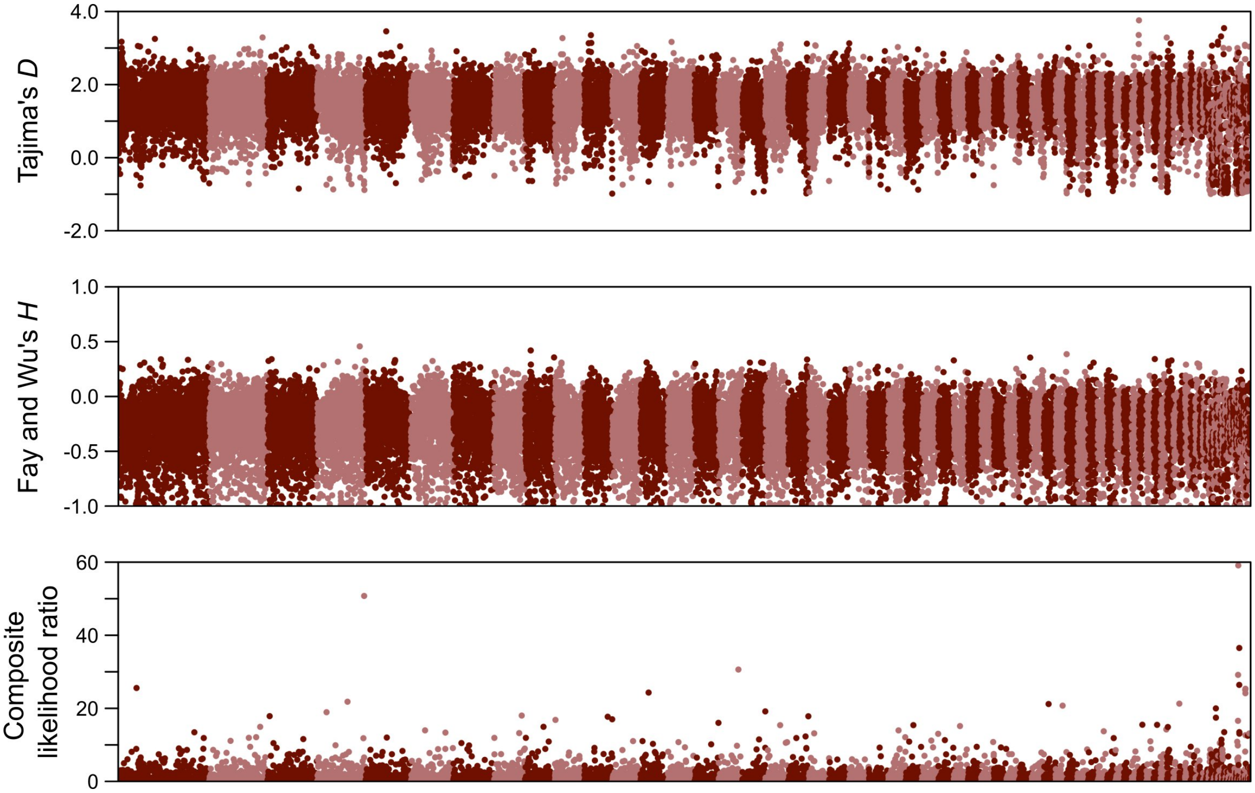

Figure S5. Manhattan plots with genome-wide scans for signatures of selection in resident white storks. Tajima's *D* (top) tests for deviations from neutrality by comparing the number of segregating sites and nucleotide diversity; Fay and Wu's *H* (middle) identifies recent selective sweeps by analysing the frequency distribution of derived alleles; the composite likelihood ratio statistic (CLR, bottom) from *SweepFinder2*, which uses the site frequency spectrum to identify loci affected by recent positive selection. The three statistics were calculated in non-overlapping 50 kb windows. Colors indicate alternating genomic scaffolds.

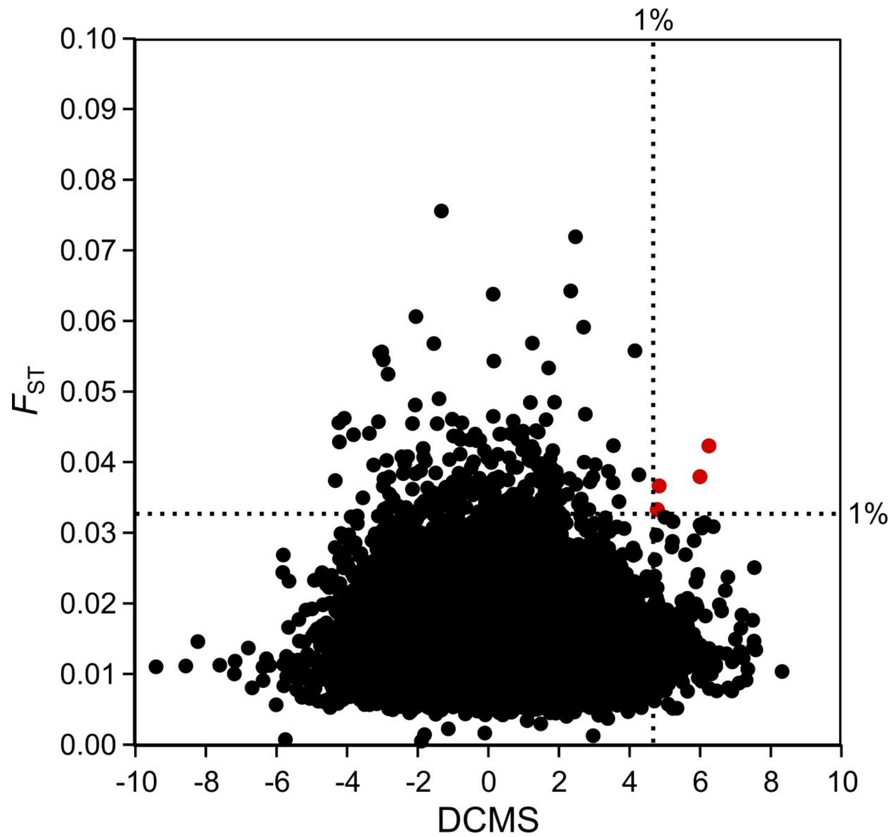

Figure S6. Comparison between values of genetic differentiation (fixation index, F_{ST}) and the de-correlated composite of multiple signals (DCMS, summarizing patterns of Tajima's *D*, Fay and Wu's *H,* and *SweepFinder2*'s composite likelihood ratio), for 22,766 genomic windows of 50 kb (non-overlapping). The four windows that are within the top 1% of both statistics are coloured in red.

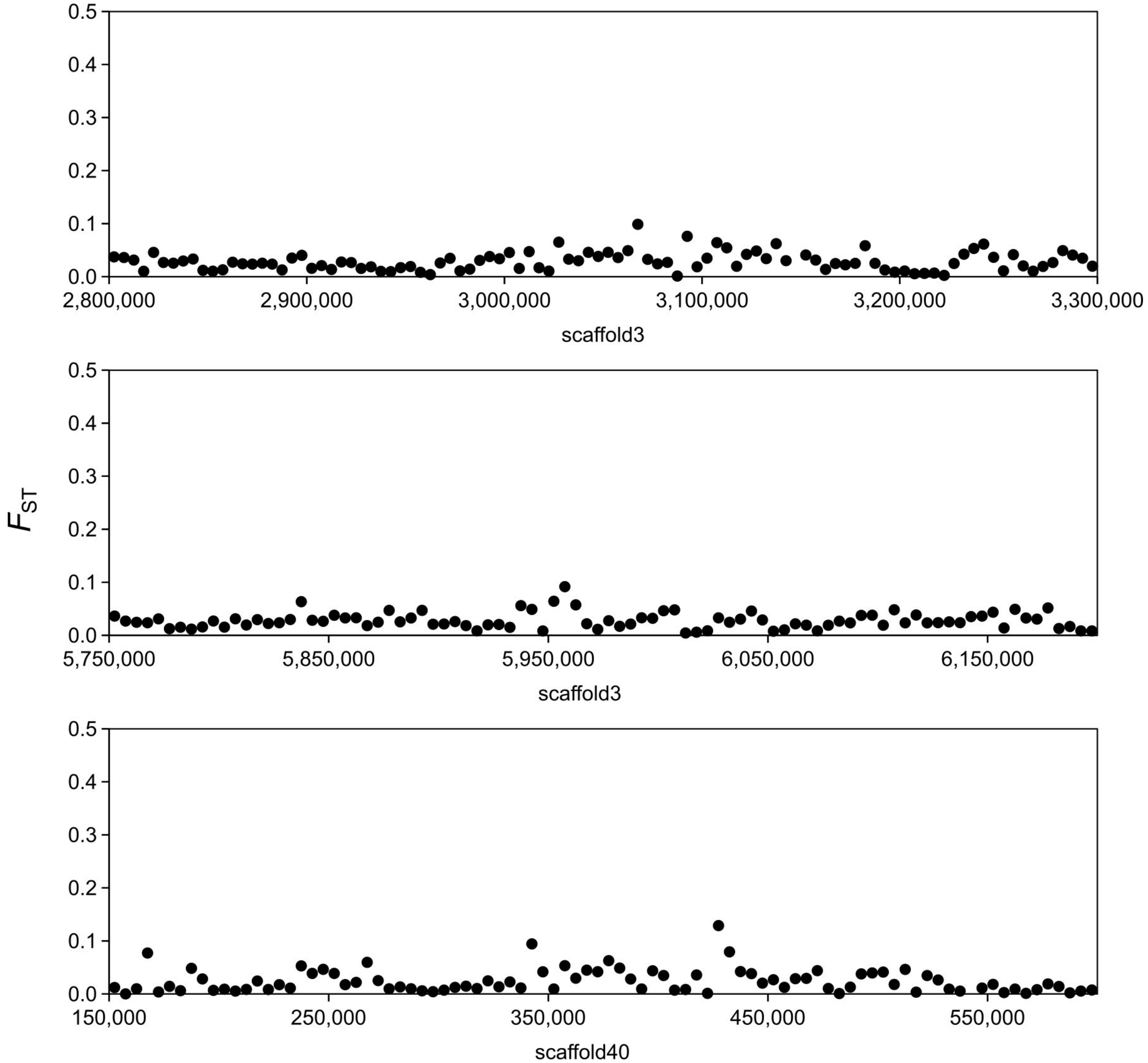

Figure S7. Genetic differentiation (fixation index, F_{ST}) between migrant and non-migrant white storks, at the three genomic regions that were 1% outliers in the F_{ST} and DCMS statistics (corresponding to the four windows in Figure S5). Each dot corresponds to a 5 kb non-overlapping window.

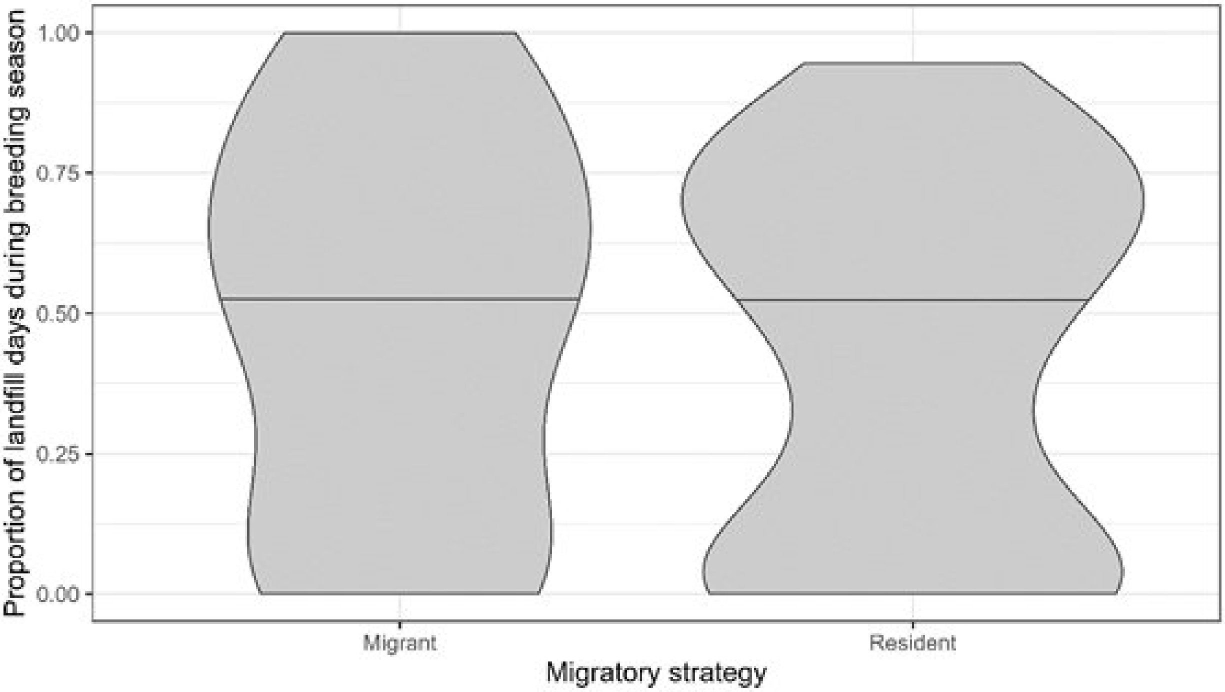

Figure S8. Proportion of landfill days during the breeding season (March, April and May) for migrant and resident white storks. The violin plot shows the distribution of the data, and the black line indicates the median proportion of landfill days of migrants and residents. Landfill days are any days in which the stork visited at least 1 landfill site. To minimize biases, the dataset includes only 1 stork-year per bird tracked for at least 60 days, during the 90 days period. This resulted in 17 migrant and 54 resident storks.

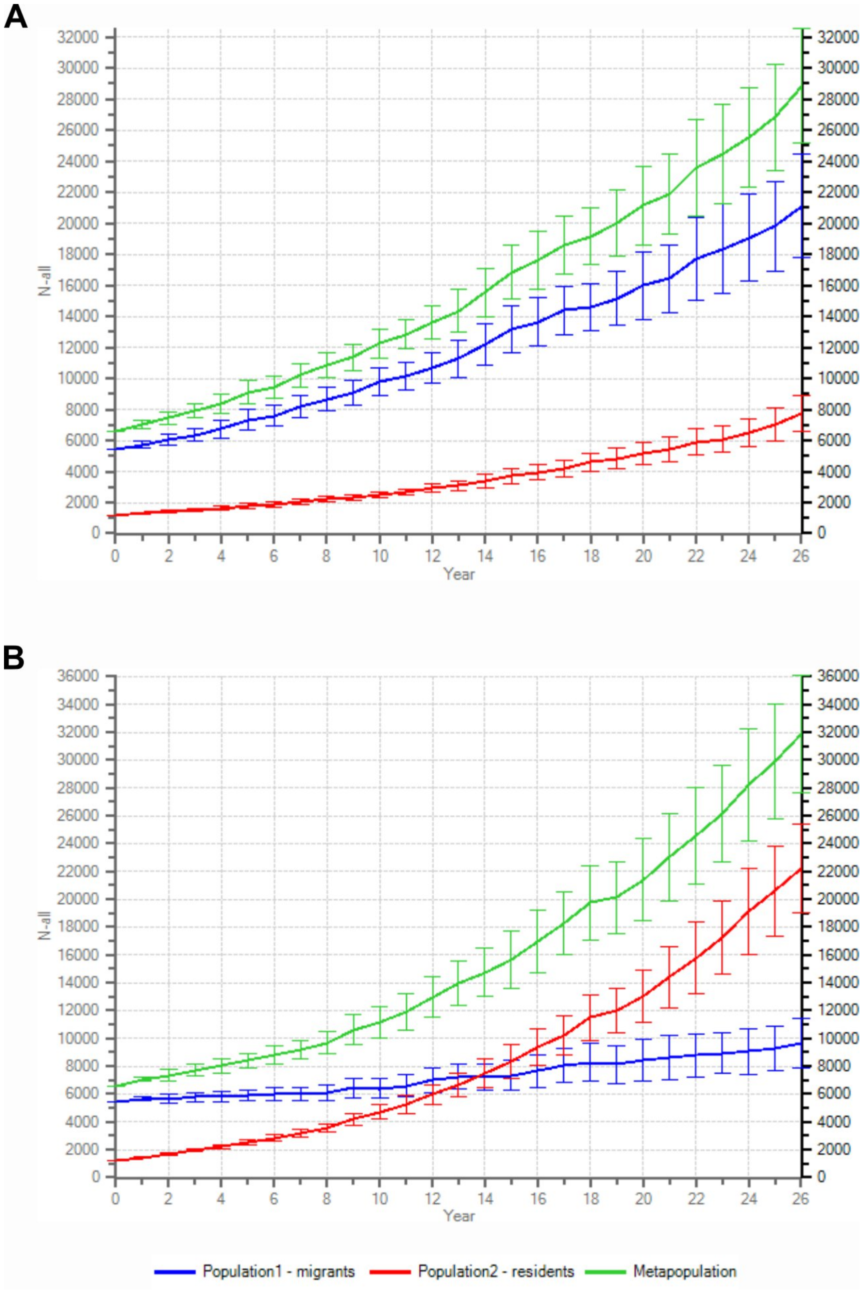

Figure S9. *Vortex10* simulations of white stork population trajectories obtained for 26 years from 1994 to 2020. The demographic parameters used are listed in Table S4. In scenario 1 (A) migratory and resident individuals do not shift migratory strategy. In scenario 2 (B) 10% of the migratory white storks (in the second and third year of life) shift to a resident strategy. The migratory population trajectory is shown in the blue line, the resident population is represented by the red line, and the metapopulation is represented in green, 95% confidence intervals are shown.

SUPPLEMENTARY TABLES

Table S1. Number of adult and juvenile white stork (*Ciconia ciconia*) GPS-tracked between 2 and 7 years. Birds tagged as juveniles that survived to adulthood are only included in the "juveniles" column.

Number of tracked years	Adults	Juveniles
2	11	13
3	17	5
4	15	3
5	3	1
6	2	1
7	1	1

Table S2. Summary statistics for the *de novo* genome assembly for the white stork *Ciconia ciconia* (Ccic_1.0). The assembly was done using 10X Genomics' Chromium linked read technology. The sample used for the assembly was an adult female, resident in Iberia (ring number MR09149, CEMPA).

	**Ccic_1.0**
Assembly size (Gb)	1.26
Scaffold N50 (Mb)	24.5
Contig N50 (kb)	300.8
Total number of scaffolds	6,992
Number of scaffolds >10 kb	890
Number of scaffolds >100 kb	165
Largest scaffold (Mb)	94.6
GC content (%)	42.1%
Complete BUSCOs reference (%)	8,091 (97.1%)
Fragmented BUSCOs reference (%)	49 (0.6%)
Missing BUSCOs reference (%)	198 (2.3%)

Table S3. Summary statistics of the whole-genome re-sequencing dataset.

Sample	Ring number (color ring)	Phenotype	Number of reads	% properly paired reads	% reads MQ>=30	Mean depth of coverage
whitestork_01	MR09459 (70+)	resident	2,832,800	95.5%	92.0%	0.3
whitestork_02	- (AE+)	resident	14,183,321	95.6%	92.8%	1.6
whitestork_03	MR07603 (AF+)	resident	21,578,092	95.6%	92.3%	2.5
whitestork_04	MR07604 (AH+)	resident	27,452,588	95.5%	92.4%	3.1
whitestork_05	MR07605 (AJ+)	migrant	26,009,892	95.1%	92.1%	2.9
whitestork_06	MR07606 (AK+)	migrant	16,392,633	95.6%	92.5%	1.9
whitestork_07	MR07607 (AL+)	resident	13,998,866	94.6%	91.1%	1.6
whitestork_08	MR07608 (AM+)	resident	25,370,010	95.2%	91.9%	2.9
whitestork_09	MR07609 (AN+)	resident	14,098,660	95.7%	92.9%	1.6
whitestork_10	MR07610 (AP+)	resident	17,334,419	95.3%	92.1%	2.0
whitestork_11	MR07611 (AR+)	migrant	9,195,414	94.9%	92.3%	1.0
whitestork_12	MR07612 (AS+)	resident	16,196,694	95.7%	92.5%	1.9
whitestork_13	MR07613 (AT+)	resident	11,430,485	96.0%	93.2%	1.3
whitestork_14	MR07614 (AU+)	resident	22,138,334	95.5%	92.7%	2.5
whitestork_16	MR07617 (AW+)	resident	24,063,455	94.9%	92.2%	2.7
whitestork_17	MR07616 (AY+)	resident	20,892,640	94.9%	91.7%	2.4
whitestork_18	MR9340 (E5+)	resident	18,629,805	94.7%	91.8%	2.1
whitestork_20	MR91330 (E7+)	resident	11,178,442	94.6%	92.1%	1.3
whitestork_21	MR9134 (E8+)	migrant	27,740,874	95.5%	92.6%	3.2
whitestork_22	MR9135 (E9+)	resident	11,331,035	94.3%	91.4%	1.3
whitestork_23	MR07602 (EA+)	resident	11,547,520	95.3%	92.2%	1.3
whitestork_25	MR09149 (EE+)	resident	23,575,502	95.8%	92.6%	2.7
whitestork_26	MR09150 (EF+)	resident	14,152,781	95.1%	92.5%	1.6
whitestork_27	- (EJ+)	migrant	28,865,537	95.6%	92.3%	3.3
whitestork_28	- (EK+)	resident	24,592,060	95.0%	91.9%	2.8
whitestork_29	MR07618 (EM+)	resident	18,765,453	95.1%	92.4%	2.1
whitestork_31	MR07620	resident	16,893,220	95.2%	92.2%	1.9

	(EP+)					
whitestork_32	MR07621 (ER+)	resident	14,205,130	95.6%	92.8%	1.6
whitestork_33	MR07622 (ES+)	resident	29,213,880	94.5%	91.4%	3.3
whitestork_34	MR07623 (ET+)	resident	19,338,640	95.0%	92.0%	2.2
whitestork_35	MR07630 (EU+)	resident	22,669,186	94.9%	92.3%	2.6
whitestork_36	MR07631 (EV+)	resident	22,316,550	94.9%	91.8%	2.5
whitestork_37	MR9136 (F0+)	resident	8,412,248	93.9%	91.4%	0.9
whitestork_39	MR9138 (F2+)	migrant	24,358,481	95.6%	92.8%	2.8
whitestork_40	MR9139 (F3+)	resident	31,316,717	95.2%	92.3%	3.6
whitestork_41	MR9140 (F4+)	resident	13,676,717	94.2%	91.7%	1.5
whitestork_42	MR9141 (F5+)	resident	11,475,570	94.3%	91.5%	1.3
whitestork_43	MR9142 (F6+)	resident	27,164,030	95.0%	91.9%	3.1
whitestork_44	MR9143 (F7+)	resident	9,320,085	94.9%	92.4%	1.1
whitestork_45	MR9144 (F8+)	resident	33,445,803	95.0%	92.0%	3.8
whitestork_46	MR9145 (F9+)	resident	18,048,925	94.2%	91.5%	2.0
whitestork_48	MR09088 (73+)	resident	10,073,314	95.2%	92.5%	1.2
whitestork_49	MR09089 (74+)	migrant	6,557,210	95.1%	94.4%	0.8
whitestork_50	MR09087 (8N+)	resident	20,539,108	95.1%	92.2%	2.3
whitestork_51	MR09086 (8M+)	resident	33,004,901	95.5%	92.3%	3.8
whitestork_52	MR09090 (AA+)	resident	15,313,648	94.6%	91.7%	1.7
whitestork_53	MR09091 (AX+)	resident	17,109,112	94.7%	91.6%	1.9
whitestork_54	MR09303 (2X+)	migrant	20,331,304	94.0%	91.3%	2.3
whitestork_55	MS03198 (K0+)	migrant	20,444,297	95.1%	92.2%	2.3
whitestork_56	MS03199 (K1+)	resident	19,911,560	94.8%	91.7%	2.2
whitestork_57	MS03200 (K2+)	resident	8,794,982	94.8%	91.9%	1.0
whitestork_58	MS02451 (K3+)	resident	6,704,429	94.2%	92.3%	0.8
whitestork_60	MS02453 (K5+)	migrant	22,828,892	96.0%	93.2%	2.6
whitestork_62	MS02500 (LP+)	migrant	15,205,512	93.6%	91.2%	1.7

Table S4. *Vortex10* parameters that were used to model the changes in the number of resident and migratory white storks in Portugal.

Parameter	Value	Sources/Supporting literature
Scenario settings		
Number of iterations	20	
Number of years	26	
Duration of each year in days	365	
Species description		
Inbreeding depression	N/A	
EV correlation between reproduction and survival	0.5	Default value
Dispersal between populations (only included in scenario 2) (Population 1 – migrants; population 2 – residents)	***Scenario 2***	
Age of individuals dispersing	2 and 3	
% of survival of dispersers	100	
% of individuals of each age class that disperse from pop. 1 to pop. 2	10	
% of individuals of each age class that disperse from pop. 2 to pop. 1	0	
Reproductive system		
Reproductive system	Long-term monogamy	Barbraud et al., 1999
Age of first offspring	3	Barbraud et al., 1999; Soriano-Redondo et al., 2023
Max. lifespan	30	Barbraud et al., 1999; Kaluga et al., 2011
Max. Age of reproduction	30	Bochenski and Jerzak, 2006
Max. Broods/year	1	Hancock et al., 1992
Max. Progeny/brood	4	Authors' unpublished data
Sex ratio at birth in % of males	50	
Density-dependence reproduction	No	
Reproductive rates		
% adult females breeding	95	Authors' unpublished data
SD in % of breeding due to EV	10	Default value
Distribution of broods per year/proportion of successful nest	0 – 5%<br>1 – 95%	Authors' unpublished data
Distribution of offspring per brood	1.7	Authors' unpublished data
SD of distribution of offspring per year	0.45	Authors' unpublished data

Mortality rates	Migrants / Residents	
Mortality (%) from age 0 to 1	65.1 / 65.1	Mayall et al., 2023; Soriano-Redondo et al., 2023
SD (%) in 0 to 1 mortality due to EV	10 / 10	Mayall et al., 2023; Soriano-Redondo et al,. 2023
Mortality from age 1 to 2	22.2 / 17.7	Mayall et al., 2023
SD in 1 to 2 mortality due to EV	3 / 3	Mayall et al., 2023
Mortality from age 2 to 3	11 / 9	Soriano-Redondo et al., 2023
SD in 2 to 3 mortality due to EV	3 / 3	Soriano-Redondo et al., 2023
Mortality after age 3	11 / 9	Soriano-Redondo et al., 2023
SD in mortality after age 3	3 / 3	Soriano-Redondo et al., 2023
Mate monopolization		
% males in the breeding pool	100	Assume all attempted to breed
Initial population size		
Population 1 – migrants	5416	Catry et al., 2017
Population 2 – residents	1180	Catry et al., 2017
Specified age distribution	Stable age distribution	default
Carrying capacity		
K	35000	
SD in K due to EV	0	

Table S5. Population demography parameters from *Vortex10* comparing different migratory strategies for the Portuguese white stork population. In scenario 1 individuals do not shift migratory strategy. In scenario 2, 10% of the migratory storks (in the second and third year of life) shift to a resident strategy. Set. r = deterministic growth rate; Stoch. r = stochastic growth rate; SE=standard error; N=population size.

***Vortex10* model**	**Description**	**Det. r**	**Stoch. r ± SE**	**N after 26 years ± SE**	**% of the metapopulation**
Scenario 1 – no dispersal	Population 1 - migrants	0.0509	0.0506 ± 0.0207	21087 ± 1600	73.1
	Population 2 - residents	0.0746	0.0703 ± 0.0201	7755 ± 550	26.9
	Metapopulation	0.0627	0.0556 ± 0.0186	28842 ± 1776	100
Scenario 2 – 10% dispersal from the migrant to the resident population	Population 1 - migrants	0.0509	0.0192 ± 0.0201	9630 ± 859	30.2
	Population 2 - residents	0.0746	0.1107 ± 0.0199	22232 ± 1537	69.8
	Metapopulation	0.0627	0.0588 ± 0.0174	31862 ± 2024	100

REFERENCES FOR SUPPLEMENTARY MATERIALS

Barbraud, C., Barbraud, J.-C., & Barbraud, M. (1999). Population dynamics of the White Stork *Ciconia ciconia* in western France. *Ibis*, *141*(3), 469-479.

BirdLife International. (2016). Species factsheet: Ciconia ciconia (2015) European Red List Assessment. [Software]. http://datazone.birdlife.org/userfiles/file/Species/erlob/supplementarypdfs/22697691_ciconia_ciconia.pdf

Brook, B. W., Cannon, J. R., Lacy, R. C., Mirande, C., & Frankham, R. (1999, February). Comparison of the population viability analysis packages GAPPS, INMAT, RAMAS and VORTEX for the whooping crane (*Grus americana*). *Animal Conservation forum.* 2(1), 23-31. https://doi.org/10.1111/j.1469-1795.1999.tb00045.x

Catry, I., Encarnação, V., Pacheco, C., Catry, T., Tenreiro, P., da Silva, L. P., & Moreira, F. (2017). Recent changes on migratory behaviour of the White stork (Ciconia ciconia) in Portugal: towards the end of migration. *Airo*, *24*, 28-35.

Hancock, J., Kushlan, J., & Kahl, P. (1992). *Storks, ibises and spoonbills of the world*. (London, UK: Academic Press.).

Kalinowski, S. T., & Hedrick, P. W. (1998). An improved method for estimating inbreeding depression in pedigrees. *Zoo Biology: Published in affiliation with the American Zoo and Aquarium Association*, *17*(6), 481-497.

Kanyamibwa, S., Schierer, A., Pradel, R., & Lebreton, J. D. (1990). Changes in adult annual survival rates in a western European population of the White Stork *Ciconia ciconia*. *Ibis*, *132*(1), 27-35.

Lacy, R. C. (2019). Lessons from 30 years of population viability analysis of wildlife populations. *Zoo biology*, *38*(1), 67-77.~

Mayall, E., Groves, L., Kennerley, R., Hudson, M., & Franco, A. (2023). Demographic consequences of management actions for the successful reintroduction of the White Stork *Ciconia ciconia* to the UK. *Bird Conservation International*, *33*, e47.

Rotics, S., Kaatz, M., Resheff, Y. S., Turjeman, S. F., Zurell, D., Sapir, N., ... & Nathan, R. (2016). The challenges of the first migration: movement and behaviour of juvenile vs. adult white storks with insights regarding juvenile mortality. *Journal of Animal Ecology*, *85*(4), 938-947.

Shephard, J. M., Ogden, R., Tryjanowski, P., Olsson, O., & Galbusera, P. (2013). Is population structure in the European white stork determined by flyway permeability rather than translocation history?. *Ecology and Evolution*, *3*(15), 4881-4895.

Soriano-Redondo, A., Franco, A. M., Acácio, M., Payo-Payo, A., Martins, B. H., Moreira, F., & Catry, I. (2023). Fitness, behavioral, and energetic trade-offs of different migratory strategies in a partially migratory species. *Ecology*, *104*(10), e4151.

Tobolka, M. (2014). Importance of juvenile mortality in Birds' population: early post-fledging mortality and causes of death in white stork *Ciconia ciconia*. *Polish Journal of Ecology*, *62*(4), 807-813.

Turjeman, S. F., Centeno-Cuadros, A., Eggers, U., Rotics, S., Blas, J., Fiedler, W., ... & Nathan, R. (2016). Extra-pair paternity in the socially monogamous white stork (*Ciconia ciconia*) is fairly common and independent of local density. *Scientific reports*, *6*(1), 27976.
